# AvaR1, a Butenolide-Type Autoregulator Receptor in *Streptomyces avermitilis*, Directly Represses Avenolide and Avermectin Biosynthesis and Multiple Physiological Responses

**DOI:** 10.3389/fmicb.2017.02577

**Published:** 2017-12-22

**Authors:** Jianya Zhu, Zhi Chen, Jilun Li, Ying Wen

**Affiliations:** ^1^State Key Laboratory of Agrobiotechnology, MOA Key Laboratory of Soil Microbiology, College of Biological Sciences, China Agricultural University, Beijing, China; ^2^Beijing Key Laboratory of Fishery Biotechnology, Beijing Fisheries Research Institute, Beijing, China

**Keywords:** *Streptomyces avermitilis*, avermectins, AvaR1, AvaR2, avenolide

## Abstract

Avermectins are commercially important anthelmintic antibiotics produced by *Streptomyces avermitilis*. The homologous TetR-family transcriptional regulators AvaR1 and AvaR2 in this species were identified previously as receptors of avenolide, a novel butenolide-type autoregulator signal required for triggering avermectin biosynthesis. AvaR2 was found to be an important pleiotropic regulator in repression of avermectin and avenolide production and cell growth, whereas the regulatory role of AvaR1 remains unclear. Investigation of AvaR1 function in the present study showed that it had no effect on cell growth or morphological differentiation, but inhibited avenolide and avermectin production mainly through direct repression of *aco* (the key enzyme gene for avenolide biosynthesis) and *aveR* (the cluster-situated activator gene). AvaR1 also directly repressed its own gene (*avaR1*) and two adjacent homologous genes (*avaR2* and *avaR3*). Binding sites of AvaR1 on these five target promoter regions completely overlapped those of AvaR2, leading to the same consensus binding motif. However, AvaR1 and AvaR2 had both common and exclusive target genes, indicating that they cross-regulate diverse physiological processes. Ten novel identified AvaR1 targets are involved in primary metabolism, stress responses, ribosomal protein synthesis, and cyclic nucleotide degration, reflecting a pleiotropic role of AvaR1. Competitive EMSAs and GST pull-down assays showed that AvaR1 and AvaR2 competed for the same binding regions, and could form a heterodimer and homodimers, suggesting that AvaR1 and AvaR2 compete and cooperate to regulate their common target genes. These findings provide a more comprehensive picture of the cellular responses mediated by AvaR1 and AvaR2 regulatory networks in *S. avermitilis*.

## Introduction

Streptomycetes have the useful capability of producing a variety of antibiotics that have broad applications in medicine and agriculture. Initiation of antibiotic biosynthesis is often accompanied by morphological development and triggered by low-molecular-weight signaling molecules (termed autoregulators) at nanomolar concentrations (Bibb, 2005). Effects of autoregulator signals are transmitted by their cognate receptor proteins, which are usually members of the TetR-family transcriptional regulators. Interaction of an autoregulator with its receptor alters the receptor's DNA-binding activity, leading to derepression of target genes involved in antibiotic biosynthesis and/or morphological differentiation (Folcher et al., 2001; Willey and Gaskell, 2011).

Twenty-four autoregulators have been described to date in 12 *Streptomyces* species, and have been classified into five groups. The three major groups are the γ-butyrolactones (GBLs), furans, and γ-butenolides. Each of the other two groups has a single member: N-methylphenylalanyl-dehydrobutyrine diketopiperazine and PI factor [2,3-diamino-2,3-bis (hydroxymethyl)-1,4-butanediol], respectively (Niu et al., 2016). The largest group is the GBLs, whose 14 members share a 2,3-disubstituted-GBL skeleton and differ in regard to stereochemistry, chain length, and branching of C2 fatty acid side chains (Choi et al., 2003; Takano, 2006). The first-described and best-studied GBL is A-factor from *Streptomyces griseus*. It binds to receptor ArpA to initiate streptomycin production and sporulation (Horinouchi and Beppu, 2007). Many *Streptomyces* genomes contain multiple genes encoding ArpA-like GBL receptor homologs, e.g., ScbR, ScbR2, CprA, and CprB in model species *S. coelicolor*; BarA and BarB in *S. virginiae*; JadR2 and JadR3 in *S. venezuelae* (Niu et al., 2016). The identified GBL/receptor pairs are SCBs/ScbR (controlling SCB and coelimycin biosynthesis) (Takano et al., 2001, 2005; Gomez-Escribano et al., 2012), VBs/BarA (controlling virginiamycin biosynthesis) (Nakano et al., 1998), and SVB1/JadR3 (controlling jadomycin biosynthesis) (Zou et al., 2014). ScbR2 and JadR2 are designated “pseudo” GBL receptors because they do not recognize GBLs; rather, they bind and respond to antibiotics as ligands to coordinate antibiotic biosynthesis (Xu et al., 2010; Wang W. et al., 2014). CprA stimulates both antibiotic production and sporulation in *S. coelicolor*, whereas CprB depresses antibiotic production (Onaka et al., 1998). BarB controls an early process of virginiamycin biosynthesis (Matsuno et al., 2004). The signals recognized by CprA, CprB, and BarB remain to be clarified.

The important industrial species *Streptomyces avermitilis* produces avermectins, which are efficient, broad-spectrum anthelmintic agents (Burg et al., 1979; Egerton et al., 1979). AveR, the only *ave* cluster-situated regulator (CSR), is essential for activation of *ave* structural genes (Guo et al., 2010). A novel γ-butenolide-type autoregulator, termed avenolide, was found to function as a signal eliciting avermectin biosynthesis at concentration 4 nM, whereas GBLs had no such effect (Kitani et al., 2011). Two butenolides (SRB1, SRB2) from *S. rochei* were subsequently identified as autoregulators that trigger lankacidin and lankamycin production (Arakawa et al., 2012). The pathway for avenolide biosynthesis remains to be fully elucidated, but has been shown to require *aco* (*sav_3706*) and *cyp17* (*sav_3704*), which respectively encode an acyl-CoA oxidase and a cytochrome P450 hydroxylase (Kitani et al., 2011). *aco*/*cyp17* homologs have been found in other species, including *S. fradiae, S. ghanaensis*, and *S. griseoauranticus*. Thus, butenolide-type autoregulators appear to be widely distributed among *Streptomyces* species, and it is of interest to elucidate their signaling cascades.

*Streptomyces avermitilis* contains an *avaR* (*S. avermitilis a*utoregulator *r*eceptors) locus that includes *avaR1* (*sav_3705*), *avaR2* (*sav_3702*), and *avaR3* (*sav_3703*). AvaR3 has an extra 75-amino acid stretch that is not present in typical GBL receptors, and promotes avermectin production through a yet-unknown regulatory mechanism (Miyamoto et al., 2011). AvaR2 is a homolog of pseudo GBL receptors ScbR2 and JadR2. Recent studies in our lab have clarified the roles of AvaR2. It is an important pleiotropic regulator of cell growth, secondary metabolism, primary metabolism, ribosomal protein synthesis, and stress responses. It inhibits avermectin and avenolide production mainly through direct repression of *aveR* and *aco*. Unlike ScbR2 and JadR2, AvaR2 binds, and responds to not only endogenous avenolide autoregulator, but also exogenous antibiotic signals that modulate its DNA-binding activity (Zhu et al., 2016). AvaR1 is a close homolog of genuine GBL receptors ArpA and ScbR. Studies by Nihira's group showed that AvaR1 acts as an avenolide receptor and binds specifically to the *aco* promoter (Kitani et al., 2011). Disruption of *avaR1* in the wild-type (WT) strain KA320 increased avenolide production, but had no effect on avermectin production (Sultan et al., 2016). On the other hand, Tang's group reported that *avaR1* deletion increased avermectin production in an avermectin high-producing strain, but had no effect on *aco* expression, and that AvaR1 did not bind to the promoter region of *aveR* (Wang J. et al., 2014). These contrasting findings for AvaR1, and our observation that AvaR2 also fuctions as an avenolide receptor, illustrate the need to further elucidate the roles of AvaR1 and its relationship with AvaR2 in *S. avermitilis*.

Results of *in vitro* and *in vivo* experiments described here clearly indicate that AvaR1 directly represses expression of *aveR* and *aco*, and thereby inhibits production of avermectin and avenolide. AvaR1 also plays a pleiotropic role in primary metabolism, ribosomal protein synthesis, stress responses, and other physiological processes. AvaR1 and AvaR2 have both common and differing target genes, and presumably cross-regulate diverse physiological processes. When AvaR1 and AvaR2 co-exist, they compete and cooperate on the same binding site in both homodimer and heterodimer form. On the basis of our findings, we propose a model in which AvaR1 and AvaR2 coordinate avermectin and avenolide biosynthesis in response to avenolide signal.

## Materials and methods

### Plasmids, strains, and growth conditions

Plasmids and strains used or constructed in the present study are listed in Table 1. *S. avermitilis* WT strain ATCC31267 was used as original host for gene manipulations. Culture conditions of *S. avermitilis* strains for avermectin production, sporulation, phenotype observation, mycelial growth, and protoplast regeneration were as described previously (Liu et al., 2015).

**Table 1:** Strains and plasmids used in this study.

**Strain or plasmid**	**Description**	**Source or reference**
***S. avermitilis***		
ATCC31267	Wild-type (WT) strain	Laboratory stock
ΔavaR1	*avaR1* deletion mutant	This study
CavaR1	*avaR1* complemented strain	This study
OavaR1	*avaR1* overexpression strain	This study
ΔavaR1/avaR1-3FLAG	*avaR1* complemented strain with AvaR1-3FLAG	This study
WT/pKC1139	WT strain containing control vector pKC1139	This study
WT/pSET152	WT strain containing control vector pSET152	This study
ΔavaR2	*avaR2* deletion mutant	Zhu et al., 2016
ΔavaR1R2	*avaR1 avaR2* double deletion mutant	This study
***E. coli***		
JM109	Routine cloning host and host for *lux* reporter system	Laboratory stock
ET12567	Non-methylating strain	MacNeil and Klapko, 1987
BL21 (DE3)	Protein overexpression host	Novagen
**Plasmids**		
pKC1139	Multiple-copy, temperature-sensitive *E. coli*-*Streptomyces* shuttle vector	Bierman et al., 1992
pSET152	Integrative *E. coli*-*Streptomyces* shuttle vector	Bierman et al., 1992
pET-28a (+)	His_6_-tagged protein expression vector	Novagen
pGEX-4T-1	GST-tagged protein expression vector	GE Healthcare
pJL117	Vector carrying ermE*p (*Streptomyces* strong constitutive promoter)	Li et al., 2010
pΔavaR1	*avaR1* deletion vector based on pKC1139	This study
pKC1139-ermp-avaR1	*avaR1* overexpression vector based on pKC1139	This study
pSET152-avaR1	*avaR1* complemented vector based on pSET152	This study
pET28-avaR1	His_6_-AvaR1 expression vector based on pET-28a (+)	Zhu et al., 2016
pIJ10500	Vector carrying *3×flag* fragment	Pullan et al., 2011
pSET152-avaR1-3FLAG	*avaR1* complemented vector with *avaR1*-*3×flag* on pSET152	This study
pCS26-*Pac*	Vector carrying promoterless *lux* reporter	Tahlan et al., 2007
pOaveRlux	pCS26-*Pac* carrying *aveR* promoter-controlled *lux* reporter	Zhu et al., 2016
pACYC184	Protein expression vector in reporter system	Tahlan et al., 2007
pAvaR1	AvaR1 expression vector in reporter system	This study
pGEX-avaR2	GST-AvaR2 expression vector based on pGEX-4T-1	This study
pET28-avaR2	His_6_-AvaR2 expression vector based on pET-28a (+)	Zhu et al., 2016

*Escherichia coli* JM109 was used for DNA cloning. *E. coli* ET12567 (MacNeil and Klapko, 1987) was used to generate non-methylated plasmids for transformation into *S. avermitilis*. Antibiotics were added as described previously (Zhao et al., 2007).

### Construction of *S. avermitilis* mutants

To construct an *avaR1* gene deletion mutant, two fragments flanking *avaR1* were amplified by PCR using WT genomic DNA as template. A 379-bp 5′ flanking region (positions −336 to +43 relative to the *avaR1* start codon) was amplified with primers ZJY107 and ZJY108, and a 368-bp 3′ flanking region (positions +645 to +1012) was amplified with primers ZJY109 and ZJY110. The two fragments were assembled by fusion PCR with primers ZJY107 and ZJY110 and cloned into pKC1139 to generate *avaR1*-deletion vector pΔavaR1, which was introduced into WT protoplasts. The desired *avaR1*-deleted mutant, termed ΔavaR1, was selected as described previously (Zhao et al., 2007), and confirmed by PCR with primer pairs ZJY115/ZJY116 (flanking the exchange regions) and ZJY117/ZJY118 (located within the deletion region) (Figure S1), followed by DNA sequencing.

For complementation of ΔavaR1, a 921-bp DNA fragment carrying the *avaR1* open reading frame (ORF) and its native promoter was obtained by PCR using primers ZJY111 and ZJY112. The PCR product was ligated into pSET152 to generate *avaR1-*complemented vector pSET152-avaR1, which was then introduced into ΔavaR1 to obtain complemented strain CavaR1. For overexpression of *avaR1*, a 708-bp fragment containing the *avaR1* ORF and a 195-bp fragment containing promoter ermE^*^p from pJL117 were ligated simultaneously into pKC1139 to produce AvaR1 overexpression vector pKC1139-ermp-avaR1, which was introduced into WT strain to construct *avaR1* overexpression strain OavaR1.

To express 3×flag-tagged AvaR1 in *S. avermitilis*, the *avaR1* gene carrying its own promoter was amplified with primers ZJY121 and ZJY122, and 3×flag fragment was amplified from pIJ10500 with primers ZJY45 and ZJY46. The resulting 993-bp *avaR1* gene and the 87-bp FLAG fragment were ligated simultaneously into pSET152 to generate pSET152-avaR1-3FLAG, which was transformed into ΔavaR1 to obtain recombinant strain ΔavaR1/avaR1-3FLAG for expression of C-terminally 3×flag-tagged AvaR1. All primers used are listed in Table S1.

To construct an *avaR1 avaR2* double deletion mutant, vector pΔavaR1 was transformed into protoplasts of *avaR2* deletion mutant ΔavaR2 (Zhu et al., 2016). The desired mutant, ΔavaR1R2, was isolated by selection of the ΔavaR1 mutant.

### Analysis of avermectin production and cell growth

Fermentation of *S. avermitilis* strains, and HPLC conditions for quantitative analysis of avermectin yield, were as described previously (Chen et al., 2007). For cell growth determination, mycelia from 50 mL cell cultures grown in soluble fermentation medium FM-II (Guo et al., 2010) were collected by centrifugation, dried at 80°C to constant weight, and weighed.

### Quantitative real-time RT-PCR (qRT-PCR) analysis

Mycelia of *S. avermitilis* WT, ΔavaR1, and ΔavaR2 grown in FM-I fermentation medium (Chen et al., 2007) were harvested at various times for RNA isolation. Extraction of total RNAs, synthesis of cDNAs, and qRT-PCR analysis of transcription levels of various genes using the primers listed in Table S1 were performed as described previously (Luo et al., 2014). Housekeeping gene 16S *rRNA* from WT was used as internal control. Gene expression was determined in triplicate.

### Western blotting

Total protein of ΔavaR1/avaR1-3FLAG was prepared from cultures grown in FM-I for various durations. Western blotting was performed as described previously (Guo et al., 2010). Mouse ANTI-FLAG mAb (M2; Sigma, USA) was used at ratio 1:5,000.

### Overexpression and purification of GST-AvaR2

To prepare GST-AvaR2 protein, the 657-bp *avaR2* coding region was amplified using primers ZJY135 and ZJY136. The PCR product was digested with *Bam*HI/*Eco*RI and cloned into expression vector pGEX-4T-1 to generate pGEX-avaR2, which was confirmed by DNA sequencing, and then transformed into *E. coli* BL21 (DE3) for overexpression of GST-tagged recombinant protein. Cells were induced with IPTG, and those containing GST-AvaR2 protein were collected, resuspended in lysis buffer (Luo et al., 2014), disrupted by sonication on ice, and centrifuged. Soluble GST-AvaR2 present in supernatant was purified with glutathione-sepharose beads (CWBIO, China) according to the manufacturer's protocol, and stored at −80°C.

### Electrophoretic mobility shift assays (EMSAs)

DNA probes carrying promoter regions of tested genes were amplified by PCR with their respective primers (Table S1). The 3′ ends of PCR products were labeled with digoxigenin (DIG) using terminal transferase, and incubated individually with various quantities of His_6_-AvaR1 and/or GST-AvaR2 in a binding reaction. Overexpression and purification of His_6_-AvaR1, and EMSA conditions, were as described previously (Zhu et al., 2016). To confirm specificity of AvaR1-probe interaction, a ~200-fold excess of nonspecific DNA or each unlabeled specific probe was added to the binding mixture before incubation.

### Chromatin immunoprecipitation-quantitative PCR (ChIP-qPCR)

Mycelia of ΔavaR1/avaR1-3FLAG cultured in FM-II for various times were harvested, and processed as described previously (Zhu et al., 2016).

### Bioluminescence detection in *E. coli*

The plasmid pOaveRlux containing *aveRp* controlled *lux* reporter genes was constructed previously in our lab (Zhu et al., 2016). For expression of AvaR1, the *avaR1* gene containing its ribosome-binding site (RBS) sequence and ORF was amplified with primers ZJY127 and ZY128. After cleavage with *Bam*HI, the 736-bp *avaR1* gene fragment was ligated into *Eco*RV/*Bam*HI-digested pACYC184 (Tahlan et al., 2007) to give pAvaR1. Control vector pACYC184 and AvaR1 expression vector pAvaR1 were separately transformed into *E. coli* strain bearing pOaveRlux. Bioluminescence of *E. coli* reporter cultures was detected as described previously (Zhu et al., 2016).

### DNase I footprinting

A fluorescence labeling procedure (Zianni et al., 2006) was used for these assays with some modifications (Zhu et al., 2016). In brief, 5′ FAM fluorescence-labeled probes corresponding to upstream regions of AvaR1 target genes were synthesized by PCR using primers listed in Table S1, and then incubated with various concentrations of His_6_-AvaR1. Followed by DNase I digestion, DNA samples were extracted and subjected to capillary electrophoresis by loading into a 3730XL DNA Genetic Analyzer. Electropherograms were analyzed using GeneMarker software program v2.2 (Applied Biosystems).

### GST pull-down assay

pGEX-avaR2 (for expressing GST-AvaR2) was introduced into *E. coli* BL21 (DE3)/pET28-avaR2 (for expressing His_6_-AvaR2) and BL21 (DE3)/pET28-avaR1 (for expressing His_6_-AvaR1) (Zhu et al., 2016), respectively. Bacteria containing pGEX-4T-1 and pET28-avaR2, or pGEX-4T-1 and pET28-avaR1, were used as negative controls. Following IPTG induction, cells containing both GST and His_6_-tagged proteins were disrupted in lysis buffer (Luo et al., 2014) by sonication on ice, and then centrifuged. The lysate containing total protein was incubated with glutathione-sepharose beads overnight at 4°C, and washed three times with PBS buffer (0.113 M NaH_2_PO_4_, 0.387 M Na_2_HPO_4_, 1.5 M NaCl). The beads were boiled with SDS sample buffer, and the eluted bound proteins were subjected to SDS-PAGE and Western blotting with anti-GST or anti-His antibody (Tiangen, China).

## Results

### AvaR1 is a negative regulator of avermectin production

**Figure 1:**
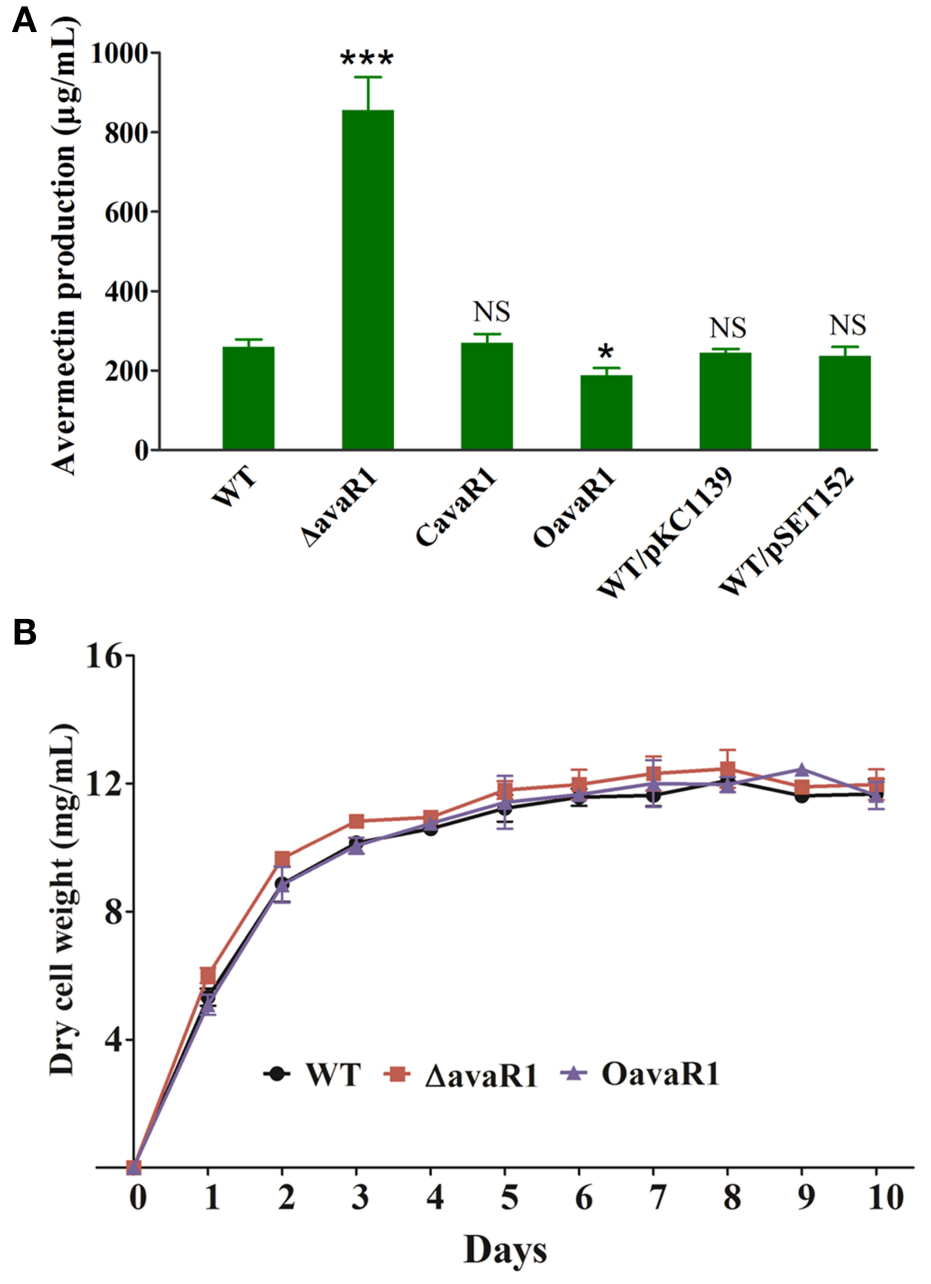
Effects of deletion and overexpression of *avaR1* on avermectin production and cell growth in *S. avermitilis*. **(A)** Comparison of avermectin production in WT, *avaR1* deletion mutant (ΔavaR1), complemented strain (CavaR1), and overexpression strain (OavaR1) cultured in FM-I for 10 days. WT/pKC1139 and WT/pSET152: vector control strains. Error bars: standard deviation (SD) from three replicate experiments. NS, not significant; ^*^, *P* < 0.05; ^***^, *P* < 0.001 for comparison with WT (Student's *t*-test). **(B)** Growth curves of WT, ΔavaR1, and CavaR1 in FM-II. Error bars: SD from three replicates.

To clarify the function of AvaR1 in *S. avermitilis*, we constructed *avaR1* deletion mutant ΔavaR1 by homologous recombination (Figure S1). HPLC analysis of fermentation products showed that avermectin yield of ΔavaR1 grown in FM-I for 10 days was ~2 fold higher than that of WT (Figure 1A). When an intact *avaR1* gene in integrative vector pSET152 was introduced into ΔavaR1, avermectin yield in complemented strain CavaR1 was restored to WT level. Overexpression of *avaR1* by introduction of plasmid pKC1139-ermp-avaR1 into WT (strain OavaR1) resulted in 30% reduction of avermectin yield. Vector control strains WT/pSET152 and WT/pKC1139 produced closely amount of avermectins to that of WT (Figure 1A). These findings indicate that AvaR1 has an inhibitory effect on avermectin production.

To assess the effect of AvaR1 on cell growth, we measured biomasses of WT, ΔavaR1, and OavaR1 cultured in FM-II. Deletion and overexpression of *avaR1* had no effect on biomass (Figure 1B), indicating that altered avermectin yield in ΔavaR1 and OavaR1 did not result from changes in growth. ΔavaR1 and OavaR1 grew normally on YMS, MM and RM14 plates (Figure S2), indicating that AvaR1 is not involved in regulation of morphological differentiation.

### AvaR1 represses expression of *ave, aco, cyp17*, and *avaR* genes

**Figure 2:**
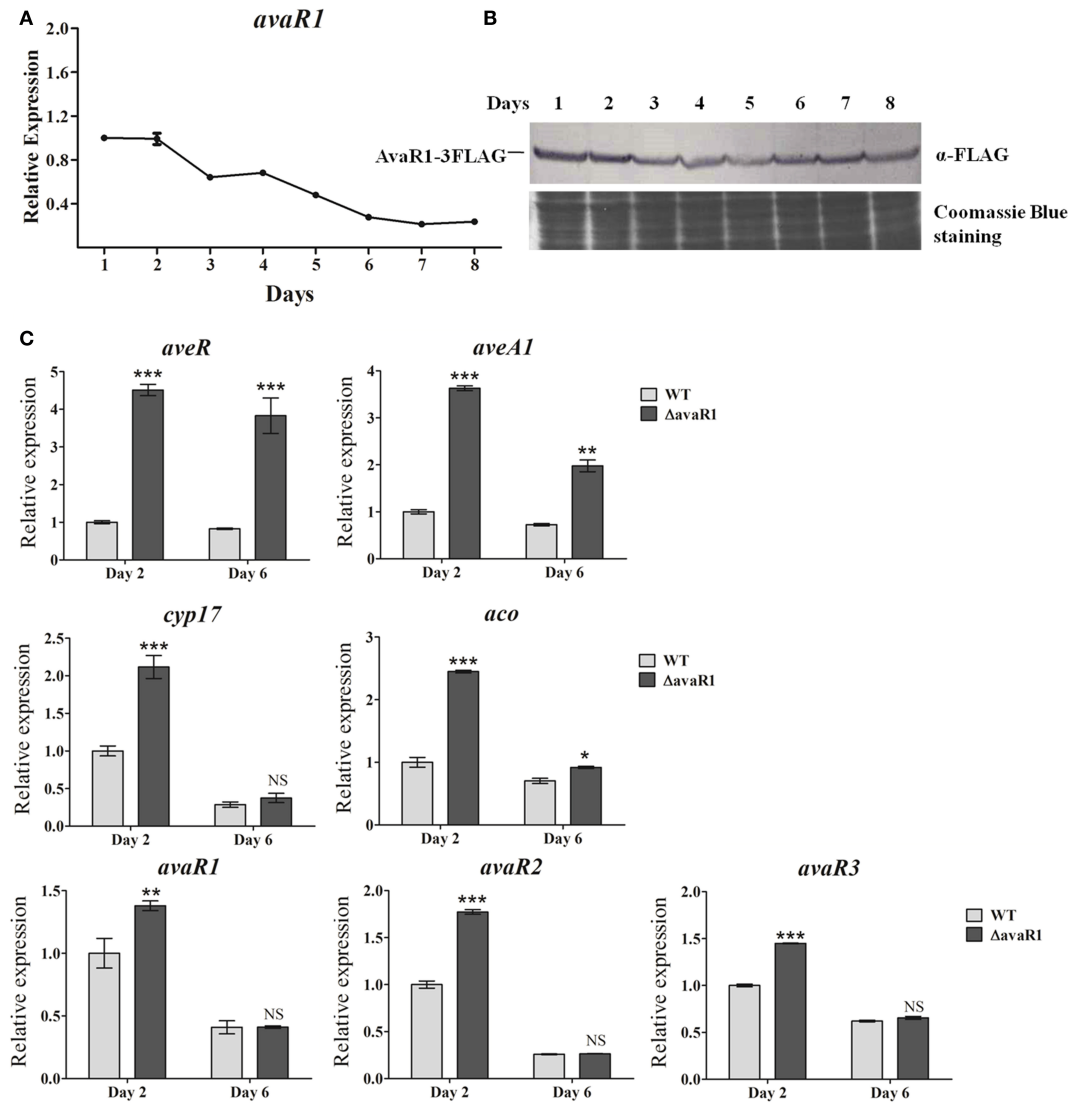
Expression analysis of *avaR1* and related genes. **(A)** Transcriptional profile of *avaR1* during avermectin production process in WT grown in FM-I. Relative value of *avaR1* on day 1 was assigned as 1. Error bars: SD from three replicates. **(B)** Western blotting analysis of AvaR1 protein expression profile during fermentation process. AvaR1 temporal expression in strain ΔavaR1/avaR1-3FLAG grown in FM-I was analyzed using ANTI-FLAG mAb. 100 μg total protein was added in each lane. Loading control: Coomassie Blue staining of total protein. **(C)** qRT-PCR analysis of *aveR, aveA1, cyp17, aco*, and three *avaR* genes in WT and ΔavaR1 grown in FM-I. Value for each gene was expressed relative to that of WT on day 2, which was assigned as 1. *avaR1*, 125-bp transcript amplified from the remainder *avaR1* ORF in ΔavaR1 with primers ZJY129 and ZJY130. Error bars: SD from three replicates. NS, not significant; ^*^, *P* < 0.05; ^**^, *P* < 0.01; ^***^, *P* < 0.001 (Student's *t*-test).

To clarify the relationship between AvaR1 and avermectin production, we examined *avaR1* expression profiles of WT cultured in FM-I. *avaR1* transcript was monitored by qRT-PCR throughout the avermectin fermentation process (Figure 2A). Transcript level was highest on day 1, remained high on day 2, then declined gradually, and was low from day 6 onward. We next examined AvaR1 expression profile by Western blotting. To prevent cross immunoreactions among homologous AvaR1, AvaR2, and AvaR3, a *3*×*flag* sequence was fused to the 3′ end of *avaR1* in integrative vector pSET152 and transformed into ΔavaR1. Expression of fusion protein AvaR1-3FLAG in ΔavaR1 restored avermectin yield (Figure S3), indicating that AvaR1-3FLAG complemented AvaR1 function, and that AvaR1 expression profile could be monitored with ANTI-FLAG mAb in recombinant strain ΔavaR1/avaR1-3FLAG. AvaR1 protein was detectable throughout the fermentation process, and its level was maximal on day 1 (Figure 2B), consistent with its transcriptional profile. These findings suggest that AvaR1 functions throughout the entire fermentation process, and plays its regulatory role mainly in the early stage.

To test the possibility that AvaR1 regulates avermectin production through *ave* genes, we performed qRT-PCR analysis using RNAs isolated from WT and ΔavaR1 cultured in FM-I at 2 days (early exponential phase) and 6 days (stationary phase). Transcription levels of CSR gene *aveR* and structural gene *aveA1* were strongly upregulated in ΔavaR1 on both days, particularly on day 2 (Figure 2C), consistent with avermectin overproduction in ΔavaR1, indicating that AvaR1 represses expression of *ave* genes.

AvaR1, AvaR2, and AvaR3 are members of the TetR-family regulators, which are typically autoregulated (Yu et al., 2010). AvaR2, AvaR3, Cyp17, and Aco have been shown to affect avermectin production in various ways (Kitani et al., 2011; Miyamoto et al., 2011; Zhu et al., 2016). We determined transcription levels of *cyp17, aco, avaR1, avaR2*, and *avaR3* by qRT-PCR. All five genes were upregulated in ΔavaR1 on day 2 (Figure 2C). On day 6, *aco* transcription was slightly increased in ΔavaR1, whereas there was no notable change in expression of *cyp17* or the three *avaR* genes. These findings indicate that AvaR1 is autorepressed and functions as a repressor of *cyp17, aco, avaR2*, and *avaR3*, mainly in the early stage of fermentation. In view of previous findings that Aco and Cyp17 are required for avenolide biosynthesis (Kitani et al., 2011), and that AvaR3 promotes avermectin production (Miyamoto et al., 2011), it is possible that elevated expression of *aco, cyp17*, and *avaR3* also contributed to enhanced avermectin production in ΔavaR1.

### AvaR1 binds specifically to upstream regions of *aveR, ac*o, and three *avaR* genes

**Figure 3:**
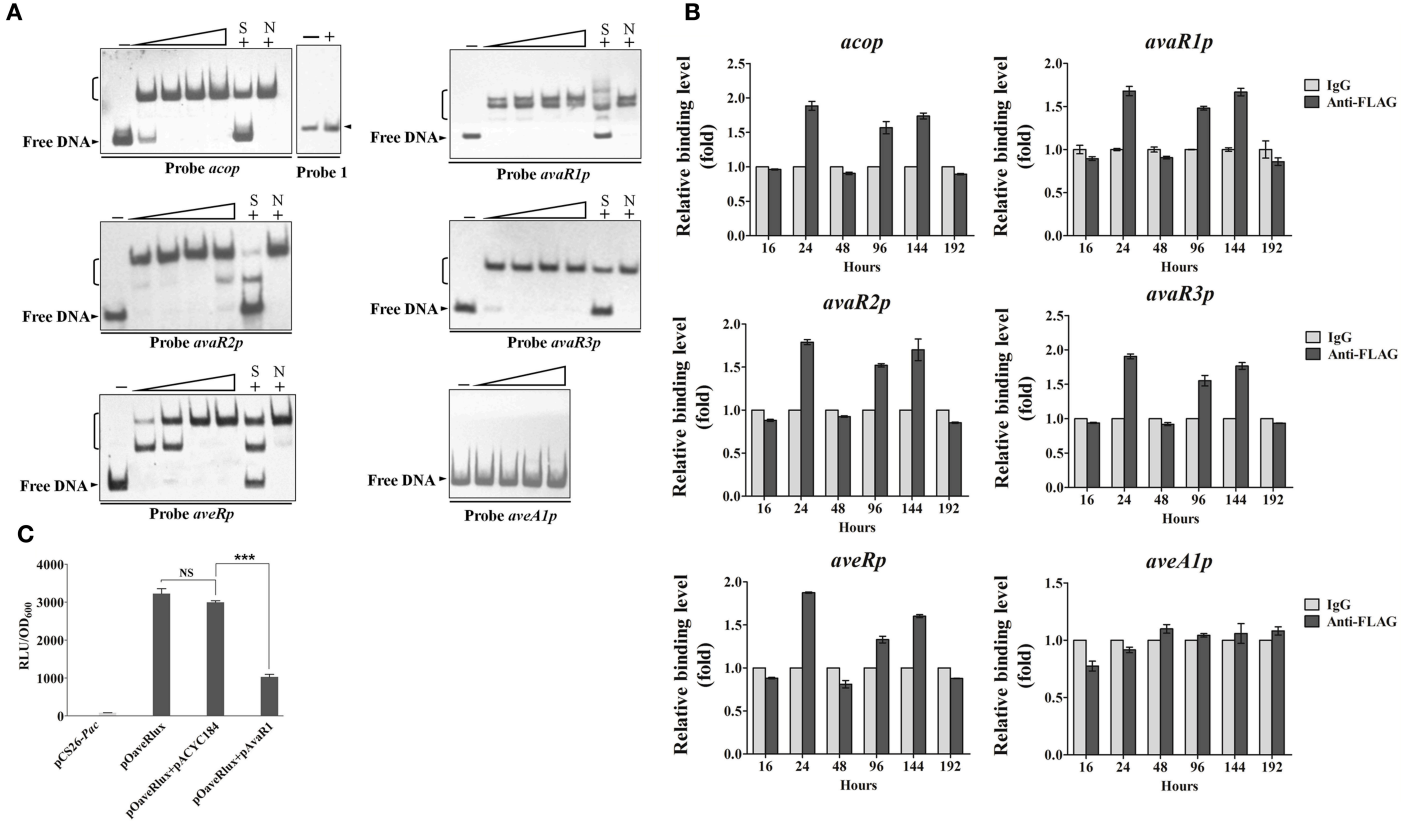
Interaction of AvaR1 with target promoters *in vitro* and *in vivo***. (A)** *In vitro* EMSAs of His_6_-AvaR1 with probes *acop, avaR1p, avaR2p, avaR3p, aveRp*, and *aveA1p* described previously (Zhu et al., 2016). Each reaction mixture contained 0.3 nM labeled probe. Specific (lanes S) and nonspecific (lanes N) competition assays were performed using ~200-fold excess of unlabeled competitor DNAs. Lanes 2–5 contained 10, 20, 50, and 100 nM His_6_-AvaR1, respectively. Lanes –: EMSAs without His_6_-AvaR1. 100 nM His_6_-AvaR1 was used for competition assays and negative control probe 1 (*sig25* promoter region) (Lanes +). Arrowheads: free probes. Brackets: AvaR1-DNA complexes. **(B)** *In vivo* ChIP-qPCR assays. ANTI-FLAG mAb against AvaR1-3FLAG was used to immunoprecipitate AvaR1-3FLAG-DNA complexes from 16-, 24-, 48-, 96-, 144-, and 192-h cultures treated with formaldehyde. IgG-coprecipitated complexes were used as negative control. Enrichment level of target DNA in control at each time point was assigned as 1. The *y* axis represents relative fold binding of target DNA compared with control. Error bars: SD from three replicate experiments. **(C)** Bioluminescence levels of *E. coli* reporter cultures containing various plasmid combinations. pCS26-*Pac* and pACYC184 were used as vector controls. Values were expressed as relative light units (RLU). Error bars: SD from three replicates. NS, not significant; ^***^, *P* < 0.001 (Student's *t*-test).

AvaR1 was reported to bind to the promoter regions of its own gene and of *aco* (Kitani et al., 2011; Sultan et al., 2016). To confirm these findings, and to determine whether the regulatory effect of AvaR1 on *aveR, aveA1, cyp17, avaR2*, and *avaR3* is direct, we performed EMSAs using soluble His_6_-AvaR1 protein (Zhu et al., 2016) and probable promoter regions of these genes. Because *cyp17* is co-transcribed with *avaR1*, we used the probes *aveRp, aveA1p, acop, avaR1p, avaR2p*, and *avaR3p*, constructed in our previous study (Zhu et al., 2016), for EMSAs. His_6_-AvaR1 did not bind to negative control probe 1, but formed complexes with probes *acop* and *avaR1p* (Figure 3A), as reported previously. His_6_-AvaR1 also bound specifically to probes *avaR2p* and *avaR3p*. No shifted band was observed for probe *aveA1p*. In contrast to the findings of Wang J. et al. (2014), probe *aveRp* generated clearly retarded signals (Figure 3A). Binding specificity was confirmed by competitive assays with a ~200-fold excess of unlabeled specific probe (lane S) and nonspecific probe 1 (lane N). These findings indicate that AvaR1 directly regulates expression of *aco, avaR1, avaR2, avaR3*, and *aveR* via binding to their promoter regions. The increased expression of *aveA1* in ΔavaR1 was presumably an indirect effect of elevated *aveR* expression. *cyp17* and *avaR1* are co-transcribed; therefore, *cyp17* is also a target gene of AvaR1, and upregulation of *cyp17* in ΔavaR1 resulted from AvaR1 autorepression.

In a search for corresponding evidence *in vivo*, we performed ChIP-qPCR experiments using samples from ΔavaR1/avaR1-3FLAG grown in FM-II for various durations, and mouse ANTI-FLAG mAb against AvaR1-3FLAG. No *aveA1p* enrichment was detected (Figure 3B), confirming that *aveA1* is indirectly regulated by AvaR1. In comparison with control *aveA1p*, AvaR1 bound to *acop, avaR1p, avaR2p, avaR3p*, and *aveRp* at 24, 96, and 144 h, with strongest binding to each of these five target promoters at 24 h (Figure 3B), consistent with the maximal expression of AvaR1 on day 1. These findings confirm dynamic binding of AvaR1 to these target promoters *in vivo*. Target DNA enrichment levels of AvaR1 were <2-fold relative to that of negative control IgG, suggesting that DNA-binding affinity of AvaR1 is low *in vivo*, and that AvaR1 is easily released from target promoters after interaction with avenolide.

To clarify the apparent discrepancy between our observed interaction of AvaR1 with *aveR* promoter region and the findings of Wang J. et al. (2014), we examined the regulatory relationship of AvaR1 with *aveR* using an *E. coli* fluorescence-reporter system (Tahlan et al., 2007). In this system, pOaveRlux was constructed previously based on pCS26-*Pac* for expressing *aveRp*-controlled *lux* reporter (Zhu et al., 2016), and pAvaR1 was constructed in this study based on pACYC184 for expressing AvaR1. pOaveRlux produced a high level of bioluminescence in *E. coli*, whereas promoterless vector pCS26-*Pac* produced only a background level (Figure 3C). Expression vector pAvaR1 clearly reduced bioluminescence of the transformant bearing pOaveRlux, whereas control vector pACYC184 had no such effect (Figure 3C). These findings are consistent with those from EMSA and ChIP-qPCR experiments, and demonstrate that AvaR1 directly represses *aveR* transcription.

### Affinity of AvaR1 binding to its target promoters

**Figure 4:**
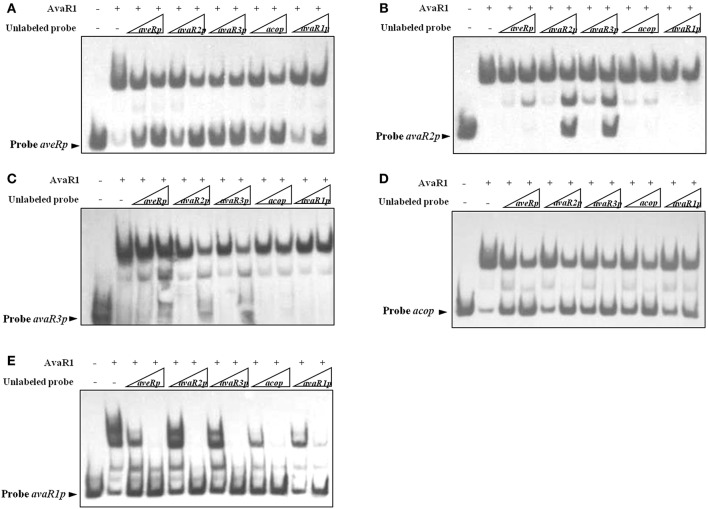
Relative affinities of AvaR1 for various target promoters. **(A)** EMSA of His_6_-AvaR1 with labeled probe *aveRp* and unlabeled probes (*aveRp, avaR2p, avaR3p, acop, avaR1p*). **(B)** EMSA of His_6_-AvaR1 with labeled probe *avaR2p* and unlabeled probes. **(C)** EMSA of His_6_-AvaR1 with labeled probe *avaR3p* and unlabeled probes. **(D)** EMSA of His_6_-AvaR1 with labeled probe *acop* and unlabeled probes. **(E)** EMSA of His_6_-AvaR1 with labeled probe *avaR1p* and unlabeled probes. For competition assays, labeled probe (0.3 nM) and unlabeled competitor probe (50- and 250-fold) were added with His_6_-AvaR1 (50 nM). Arrowheads: free labeled probes.

To compare affinity of AvaR1 for its five target promoters (*acop, avaR1p, avaR2p, avaR3p, aveRp*), we performed competitive EMSAs using labeled probes and excess unlabeled probes (specific competitor). In experiments using 50-fold excess of unlabeled probes, the dissociation of AvaR1 from labeled *aveRp* caused by unlabeled probe *avaR1p* was less than that of the other four unlabeled probes (Figure 4A). Further analysis using labeled probes *avaR2p, avaR3p*, and *avaR1p* (Figures 4B,C,E) confirmed that affinity of AvaR1 to *avaR1p* was the lowest among the five DNA targets. In experiments using 250-fold excesses, unlabeled probes *avaR3p* and *avaR2p* dissociated most AvaR1 from labeled *avaR2p*, whereas the other three unlabeled probes had little or no such effect (Figure 4B), indicating a higher relative affinity of AvaR1 for *avaR3p* and *avaR2p*. The competitive ability of 50-fold excess of unlabeled probe *avaR3p* was slightly higher than that of the same excess of *avaR2p* (Figure 4B). These findings indicate that AvaR1 had the strongest affinity for *avaR3p*. Comparison of intensities of AvaR1-*avaR3p* complex corresponding to unlabeled probes *aveRp and acop* (Figure 4C) indicated AvaR1 affinity in the order *aveRp* > *acop*. There were no striking differences of intensities of AvaR1-*acop* complex among the five unlabeled probes (Figure 4D). The above findings, taken together, indicate that binding affinity of AvaR1 to its target promoters is in the order *avaR3p* > *avaR2p* > *aveRp* > *acop* > *avaR1p*; however, the differences for *avaR3p* vs. *avaR2p*, and *acop* vs. *avaR1p*, are slight.

### Identification of precise AvaR1 binding sites

**Figure 5:**
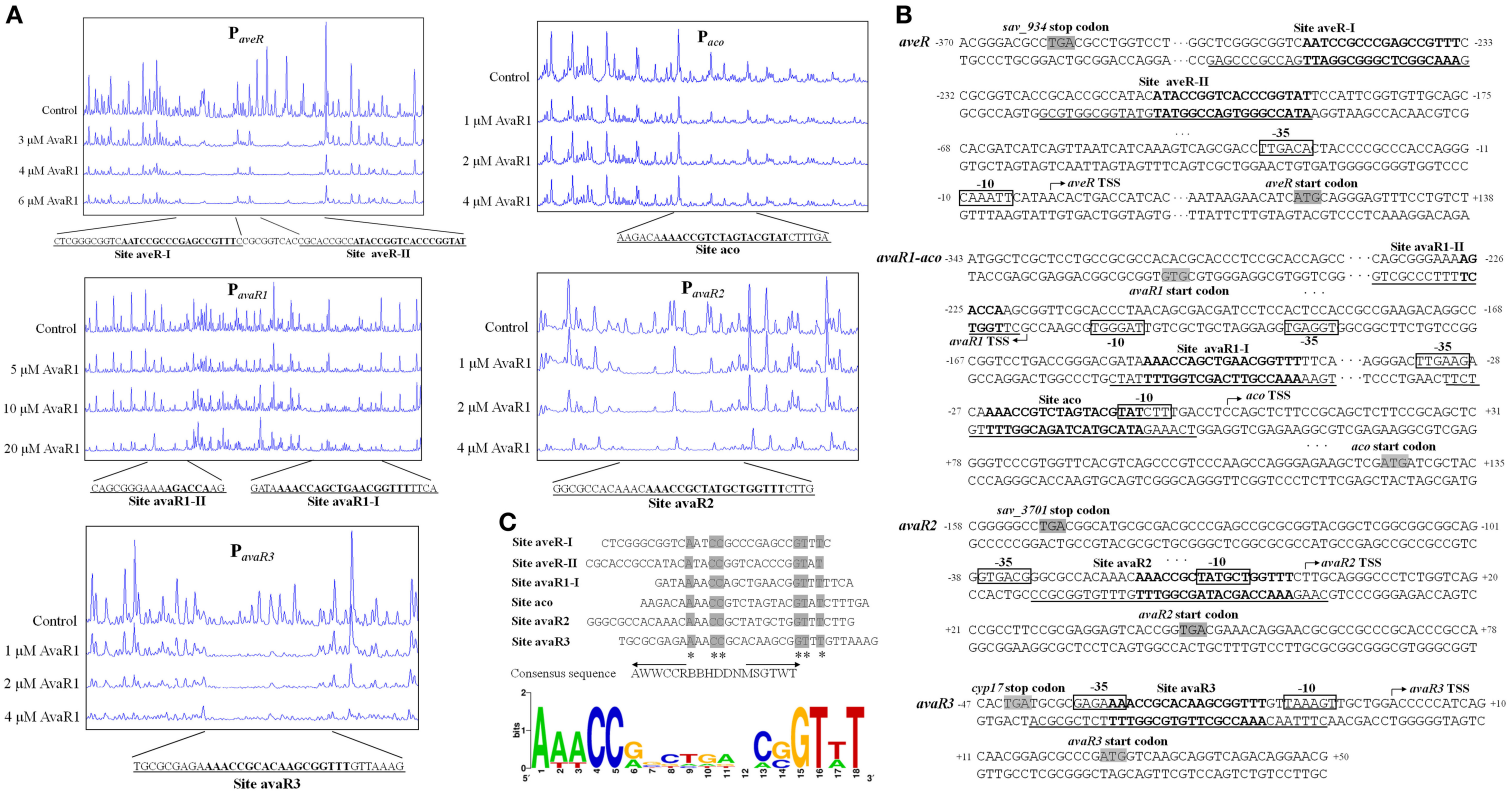
Identification of AvaR1 binding sites. **(A)** DNase I footprinting assay of AvaR1 on target promoter regions. Protection fluorograms were acquired with increasing amounts of His_6_-AvaR1. Top fluorograms: control reactions with 10 μM BSA. **(B)** Nucleotide sequences of target promoter regions and AvaR1 binding sites. Numbers: distance (nt) from respective TSS. Shaded areas: translational start codons. Bent arrows: TSSs. Boxes: potential −10 and −35 regions. Solid lines: AvaR1 binding sites. Boldface: ARE-like sequences. **(C)** Analysis of consensus AvaR1 binding sequence using the WebLogo program (http://weblogo.berkeley.edu). Asterisks: consensus bases. Arrows: inverted repeats. Height of each letter is proportional to appearance frequency of corresponding base.

The transcriptional start sites (TSSs) of *aveR, aco*, and three *avaR* genes were determined previously (Zhuo et al., 2010; Miyamoto et al., 2011; Sultan et al., 2016; Zhu et al., 2016). We performed DNase I footprinting assays to identify precise AvaR1 binding sites and elucidate the mechanism whereby AvaR1 regulates its five targets. AvaR1 was found to protect two sites (aveR-I, aveR-II), seperated by 9 nt, on the *aveR* promoter region (Figure 5A). These sites are far upstream of the *aveR* TSS. Site aveR-I corresponds to positions −262 to −233 nt, and site aveR-II −223 to −193 nt, relative to the the *aveR* TSS (Figure 5B). The mechanism whereby AvaR1 represses *aveR* is unkown. Interactions among AvaR1 and the two binding sites may prevent RNA polymerase acess to the *aveR* promoter, or recruit other repressors. The protected site of AvaR1 on the *aco* promoter region (site aco) overlaps the putative −10 and −35 promoter regions, and extends from −31 to −4 nt relative to the *aco* TSS (Figures 5A,B), suggesting that AvaR1 directly represses *aco* by impeding RNA polymerase binding to the *aco* promoter. *avaR1* promoter region was protected at two sites: site avaR1-I (positions −93 to −68 nt relative to the *avaR1* TSS) and site avaR1-II (+19 to +2 nt) (Figures 5A,B). AvaR1 protected a region from −32 to +3 nt relative to the *avaR2* TSS on the *avaR2* promoter region (site avaR2), and from −41 to −8 nt relative to the *avaR3* TSS on the *avaR3* promoter region (site avaR3) (Figures 5A,B). Site avaR1-I is located upstream of the potential −35 region of the *avaR1* promoter, and sites avaR1-II, avaR2, and avaR3 are close to or overlap the potential −10 region of their respective promoters, suggesting that AvaR1 represses three *avaR* genes through mechanisms analogous to those for *aveR* and *aco* repression.

ARE (*a*uto*r*egulatory *e*lement) sequences are found in genuine and pseudo GBL receptor binding sites in many *Streptomyces* species (Wang et al., 2011; Willey and Gaskell, 2011). AvaR1 is a receptor of γ-butenolide-type avenolide, not of GBLs; however, the protected sites of AvaR1 on the five promoter regions all contain ARE-like sequences. Site avaR1-II contains a half-length ARE, and the other sites contain a full-length ARE. Interestingly, the binding sites of AvaR1 completely overlap those of AvaR2 on the five target promoter regions (Figure S4; Zhu et al., 2016), with the same protection sequences on *aveRp, acop*, and *avaR1p*, and three or four nucleotides more than the AvaR2 binding sequences on *avaR3p* and *avaR2p*. WebLogo program analysis of AvaR1 binding sites including full-length AREs produced an 18-bp consensus ARE-like palindromic sequence (5′-AWWCCRBBHDDNMSGTWT-3′) (Figure 5C), identical to that of the AvaR2 consensus binding motif (Figure S4; Zhu et al., 2016).

### AvaR1 and AvaR2 have both common and exclusive target genes

**Figure 6:**
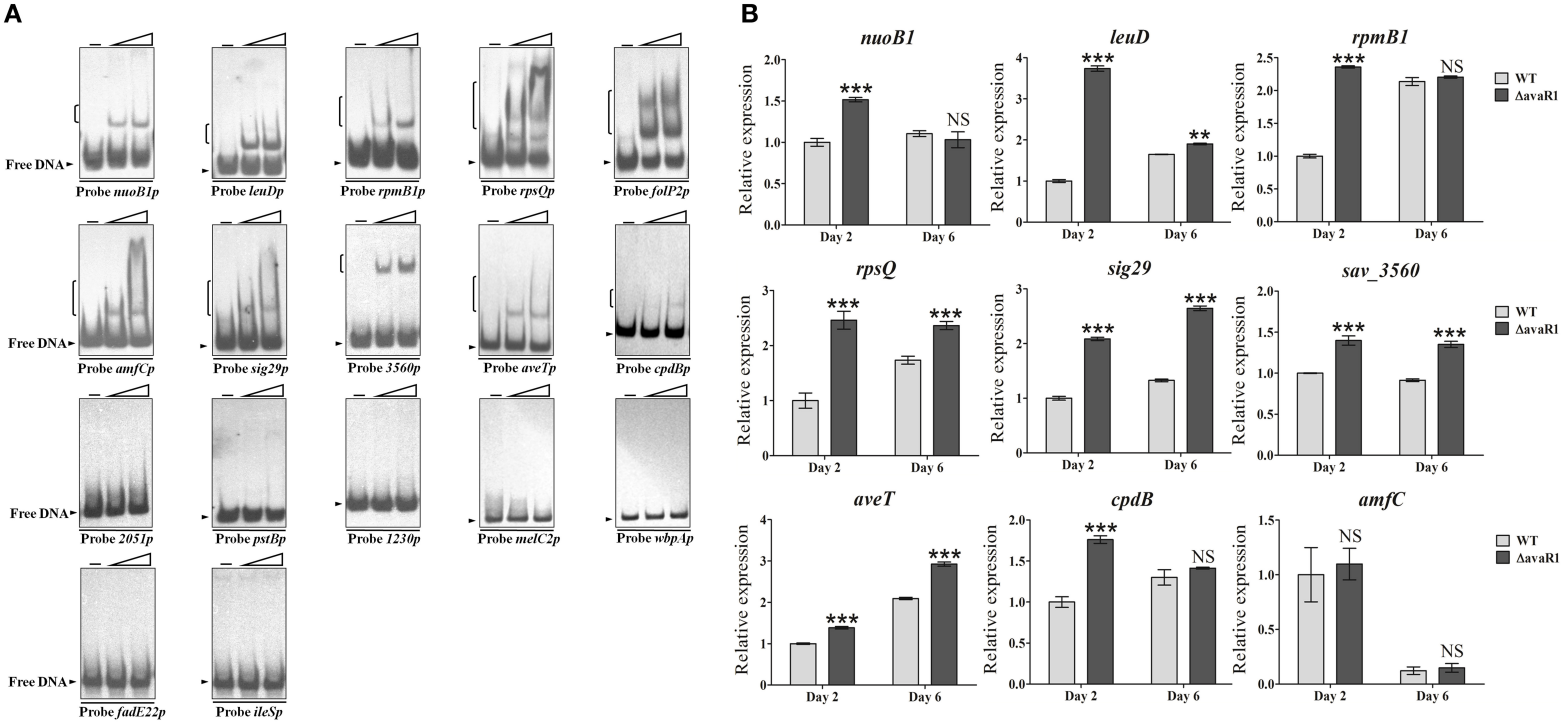
Confirmation of new AvaR1 target genes. **(A)** EMSAs of His_6_-AvaR1 protein with 17 putative binding promoter regions. Each lane contained 0.3 nM labeled probe. Lanes –, EMSAs without His_6_-AvaR1. Lanes 2 to 3 contained 50 and 200 nM His_6_-AvaR1, respectively. **(B)** qRT-PCR analysis of newly identified AvaR1 target genes in WT and ΔavaR1 strains. WT value of each gene on day 2 was assigned as 1. Error bars: SD from three replicates. NS, not significant; ^**^, *P* < 0.01; ^***^, *P* < 0.001 (Student's *t*-test).

AvaR1 and AvaR2 have an identical consensus binding motif, and are therefore expected to have an identical predicted regulon based on use of the same web-based application. Because the consensus binding motif of AvaR1 and AvaR2 was deduced only from five common target promoter regions, we could not rule out the possibility that AvaR1 and AvaR2 have different targets. To test the possibility that AvaR1 binds to other AvaR2 targets in addition to the above five common targets, 11 identified AvaR2 target genes (*nuoB1, leuD, rpmB1, rpsQ, folP2, amfC, sig29, sav_3560, sav_2051, pstB*, and *sav_1230*) (Zhu et al., 2016) were subjected to EMSAs with purified His_6_-AvaR1 protein. AvaR1 bound to the promoter regions of *nuoB1, leuD, rpmB1, rpsQ, folP2, amfC, sig29*, and *sav_3560*, but not to those of *sav_2051, pstB*, or *sav_1230* (Figure 6A), indicating that AvaR1 and AvaR2 have both common and exclusive targets. To follow up on this finding, *melC2, wbpA, fadE22, ileS, aveT* (*sav_3619*), and *cpdB* listed in putative AvaR2 regulon (Zhu et al., 2016) were subjected to EMSAs with His_6_-AvaR1. Among these genes, *melC2, wbpA, fadE22*, and *ileS* were found not to be AvaR2 targets (Zhu et al., 2016). *aveTp* and *cpdBp* had the ability to interact with AvaR1, but the other four promoter probes did not (Figure 6A). *aveT* encodes a TetR-family transcriptional activator for avermectin production and morphological differentiation (Liu et al., 2015), and *cpdB* encodes a putative 2′,3′-cyclic-nucleotide 2′-phosphodiesterase. We performed EMSAs to test the possibility that AvaR2 binds to these two novel AvaR1 targets, and observed that it bound to *aveTp*, but not to *cpdBp* (Figure S5A).

The regulatory roles of AvaR1 in expression of the 10 newly identified target genes were evaluated by qRT-PCR analysis. AvaR1 repressed transcription of *nuoB1, leuD, rpmB1, rpsQ, sig29, sav_3560, aveT*, and *cpdB*; i.e., transcription levels of these targets were higher in ΔavaR1 than in WT on day 2, or on days 2 and 6 (Figure 6B). *amfC* encodes an aerial mycelium-associated protein (Yonekawa et al., 1999). *amfC* transcript levels were very similar in WT and ΔavaR1, consistent with the absence of morphological differences between these two strains. *folP2* expression was not detected in either WT or ΔavaR1 on day 2 or 6 under our fermentation conditions. The effect of AvaR2 on *aveT* expression was also examined by qRT-PCR. AvaR2 activated transcription of *aveT* (Figure S5B), in contrast to the repressing effect of AvaR1 on *aveT* (Figure 6B).

Taken together, the identified common targets of AvaR1 and AvaR2 are *aveR, aco, avaR1, avaR2, avaR3, nuoB1, leuD, folP2, rpmB1, rpsQ, amfC, sig29, sav_3560*, and *aveT. cpdB* is a target of AvaR1, but not of AvaR2. *sav_2051, pstB* and *sav_1230* are targets of AvaR2, but not of AvaR1. The above targets are involved in secondary metabolism, primary metabolism, morphological differentiation, ribosomal protein synthesis, stress responses, and other processes (Liu et al., 2015; Zhu et al., 2016), indicating that AvaR1 and AvaR2 cross-regulate a wide range of physiological processes.

Avermectin production level was much higher in single deletion mutant ΔavaR1 than in ΔavaR2, and levels in double deletion mutants ΔavaR1R2-1,-2, and -3 were intermediate (Figure S6), consistent with the finding that AvaR1 and AvaR2 have different target genes, which may affect avermectin production in different ways.

### AvaR1 and AvaR2 compete and cooperate on the same binding site

**Figure 7:**
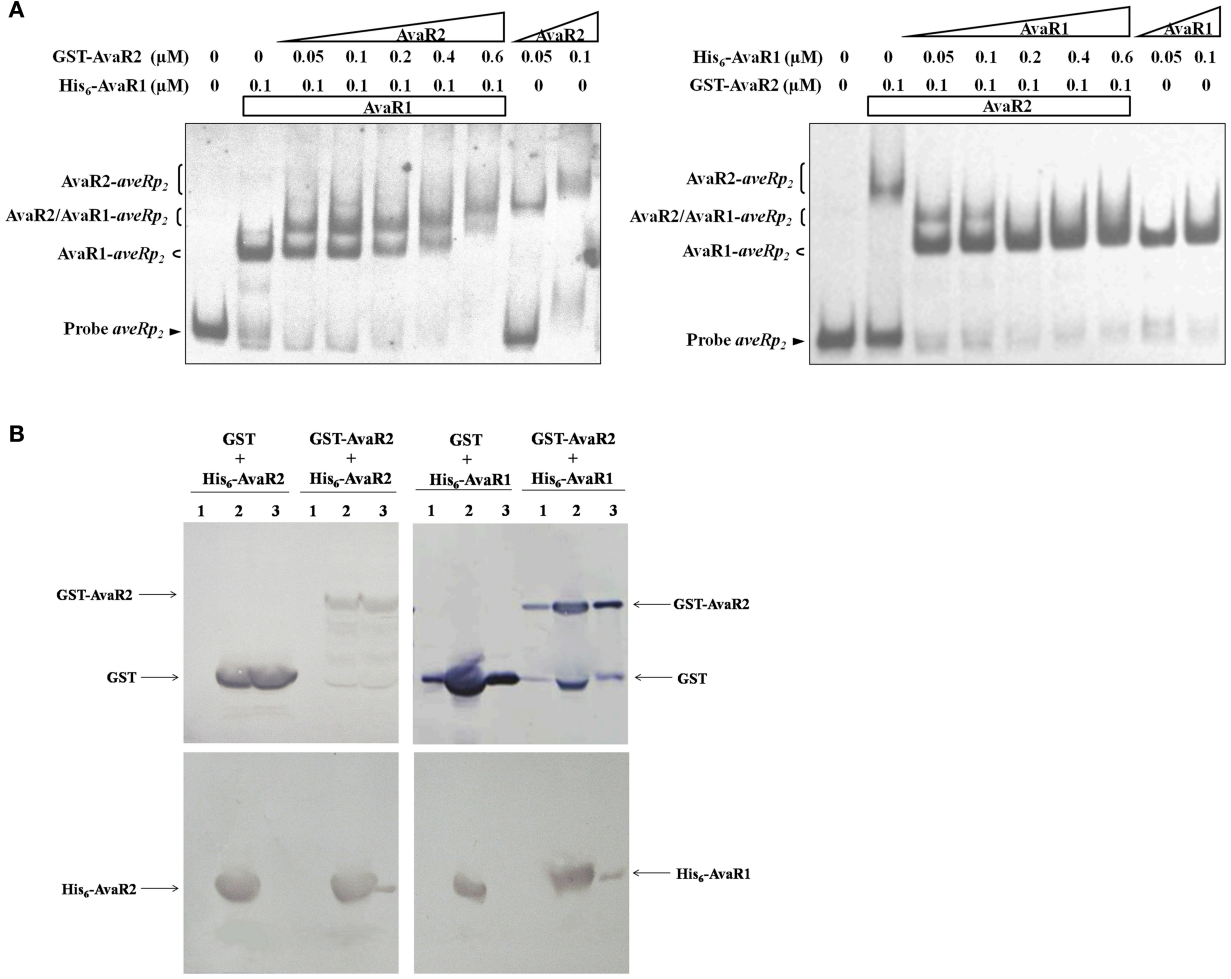
Relationships between AvaR1 and AvaR2. **(A)** Competitive EMSAs of probe *aveRp*_2_ with His_6_-AvaR1 and GST-AvaR2 proteins. 0.3 nM labeled probe *aveRp*_2_ was incubated with the indicated concentrations of His_6_-AvaR1 and GST-AvaR2. **(B)** GST pull-down assays of AvaR1 and AvaR2 from *E. coli* whole cell lysate. His_6_- and GST-tagged proteins were co-expressed in *E. coli*, recovered by sonication and centrifugation, and subjected to GST pull-down and Western blotting analysis with anti-GST and anti-His antibodies, respectively. Lanes 1: cell lysate before induction by IPTG. Lanes 2: cell lysate after induction. Lanes 3: GST pull-down.

AvaR1 and AvaR2 are TetR-family transcriptional regulators, which generally function as homodimers to bind to palindromic sequences (Yu et al., 2010). Because the two proteins are homologs, it is conceivable that they could form a heterodimer. Because they have common target genes, they may also compete or cooperate for DNA binding. The apparent molecular weights of His_6_-AvaR1 and His_6_-AvaR2 were similar (~35 kDa). To evaluate possible competition or cooperation of AvaR1 and AvaR2 on the same binding site, we expressed and purified GST-tagged AvaR2 protein from *E. coli* to separate the complexes formed by His_6_-AvaR1 or GST-AvaR2 using the same DNA probe in EMSAs. His_6_-AvaR1 and GST-AvaR2 were added both separately and together with probe *aveRp*_2_, which contains only one palindromic sequence: site aveR-II. When applied separately, both proteins retarded *aveRp*_2_ (Figure 7A). In the presence of 0.1 μM His_6_-AvaR1, an increase of GST-AvaR2 concentration resulted in reduction of AvaR1-*aveRp*_2_ complex and formation of a new complex, located between AvaR1-*aveRp*_2_ and AvaR2-*aveRp*_2_ (Figure 7A, left), which was most likely formed by AvaR1/AvaR2 heterodimer with probe *aveRp*_2_. When GST-AvaR2 was added at a concentration of 0.6 μM, the complex AvaR2-*aveRp*_2_ became predominant, and the above-mentioned new complex and AvaR1-*aveRp*_2_ disappeared (Figure 7A, left). In the presence of 0.1 μM GST-AvaR2, an increase of His_6_-AvaR1 concentration resulted in disappearance of AvaR2-*aveRp*_2_ complex and appearance of AvaR1-*aveRp*_2_ and a new band, most likely AvaR1/AvaR2-*aveRp*_2_ (Figure 7A, right). As His_6_-AvaR1 concentration increased, intensity of the new band declined and that of AvaR1-*aveR2p* became stronger. When His_6_-AvaR1 was added at a concentration of 0.2 μM, the new band disappeared and AvaR1-*aveRp*_2_ complex became predominant (Figure 7A, right). These findings suggest that AvaR1 and AvaR2 not only compete for the same DNA site, but also cooperate as a heterodimer for DNA binding.

To assess possible interaction between AvaR1 and AvaR2 under physiological conditions, we co-expressed His_6_-AvaR2 or His_6_-AvaR1 with GST or GST-AvaR2 in *E. coli*, and performed *in vivo* GST pull-down experiments. His_6_-AvaR2 was pulled down by GST-AvaR2, but not by GST (Figure 7B, left), indicating that AvaR2 interacts with itself *in vivo*, most likely to form a homodimer. His_6_-AvaR1 was also pulled down by GST-AvaR2, but not by GST (Figure 7B, right), supporting the conclusion from experiments shown in Figure 7A that AvaR1 interacts directly with AvaR2 to form a heterodimer.

## Discussion

Autoregulator/receptor regulatory systems are widespread among *Streptomyces* species, and play key roles in eliciting secondary metabolites or/and morphological differentiation (Bibb, 2005; Niu et al., 2016). Unlike typical GBL signaling molecules applied by many other *Streptomyces* species, *S. avermitilis* uses avenolide, a γ-butenolide autoregulator, to trigger avermectin production (Kitani et al., 2011). AvaR1 was shown to be an avenolide receptor (Kitani et al., 2011); however, its role in regulation of avermectin biosynthesis remains unclear. In this study, we elucidated the molecular mechanism underlying this role, and demonstrated that AvaR1 is a direct repressor of avenolide and avermectin biosynthesis as well as other physiological processes including primary metabolism, ribosomal protein synthesis, and stress responses.

AvaR1 strongly inhibits avermectin production in WT strain ATCC31267. Results of our qRT-PCR, EMSA, and ChIP-qPCR analyses revealed that this inhibitory effect is due mainly to direct repression of the cluster-situated activator gene *aveR*. Our findings are in contrast to reports by other groups that AvaR1 indirectly controls *aveR* expression (Wang J. et al., 2014), and that AvaR1 has no effect on avermectin production in WT strain KA320 (Sultan et al., 2016). Our DNase I footprinting assays showed that AvaR1 binds to two sites on the far upstream region of *aveR*: one extending from −262 to −233 nt, the other from −223 to −193 nt, relative to the *aveR* TSS. In the EMSA studies by Wang J. et al. (2014), the *aveR* promoter probe used extended from positions −109 to +113 nt relative to the *aveR* TSS, and did not contain two AvaR1-binding sites. This explains their finding that AvaR1 did not bind to the *aveR* promoter region. The differences between our findings and those of Nihira's group regarding effect of AvaR1 on avermectin production may be due to differences in experimental strains and growth conditions used. Nihira's group used KA320 as WT strain (Sultan et al., 2016), and described it as being isogenic to *S. avermitilis* MA-4680–also known as ATCC31267, the WT strain used in our study. However, in a previous report, they described KA320 as being phenotypically unstable and requiring frequent reisolation (Kitani et al., 2009). In contrast, strain ATCC31267 we used was stable throughout our experiments, and AseI restriction patterns of our ATCC31267 chromosome were identical to the published data (Guo et al., 2010). Properties of CSR gene *aveR* also differed between the WT strains used by our group and Nihara's. In our 2010 study (Guo et al., 2010), overexpression of *aveR* promoted avermectin production in ATCC31267, in accordance with the general rule that overexpression of a cluster-situated activator gene leads to increased production of the corresponding antibiotic. In contrast, the 2009 study by Nihara's group found that addition of *aveR* abolished avermectin production in KA320 and its derivative K139 (Kitani et al., 2009). It therefore seems likely that differences in genetic background exist between KA320 and our ATCC31267, which account for the different findings. Genome resequencing of the two strains can clarify such genetic differences.

The *aco* gene encodes a key enzyme for avenolide production (Kitani et al., 2011). In the present study, AvaR1 directly repressed *aco* expression, consistent with previous findings by Nihira's group (Kitani et al., 2011; Sultan et al., 2016). Wang et al. (2015) confirmed the role of AvaR1 as a direct repressor of *aco* using a genetic biosensor. AvaR1 also inhibits transcription of *cyp17*, which is involved in avenolide production. Thus, AvaR1 is an important regulator in control of avenolide level. Our 2016 study showed that AvaR2 is also a key repressor of avenolide production (Zhu et al., 2016). The increase of *aco* transcription level was >130-fold in *avaR2* deletion mutant ΔavaR2 (Zhu et al., 2016), but <2-fold in *avaR1* deletion mutant ΔavaR1, indicating that AvaR2 plays a role dominant over that of AvaR1 in control of avenolide production.

AvaR1 and AvaR2 are homologs, both act as receptors of avenolide signal (Kitani et al., 2011; Zhu et al., 2016), and bind to the same sequence on *aveR* and *aco* promoter regions. We therefore investigated possible interaction or cooperation between these two proteins to regulate avermectin and avenolide production. Competitive EMSAs and GST pull-down assays revealed coexistence of AvaR1/AvaR2 heterodimer with AvaR1 and AvaR2 homodimers. The EMSA results also showed that the heterodimer-DNA complex reduced or disappeared when concentration of one protein was much higher than that of another protein, suggesting that interconversion among AvaR1/AvaR2 heterodimer and the AvaR1 and AvaR2 homodimers is a highly dynamic process. Analogously, Li et al. (2015, 2017) demonstrated that ScbR and ScbR2 share some common binding sites, and can form a heterodimer in addition to their respective homodimers. These examples of heterodimer formation between homologous TetR-family regulators indicate that regulatory mechanisms in this family are complex, and that the heterodimers may play regulatory roles of which the corresponding homodimers are not capable. For example, ScbR/ScbR2 heterodimer has an exclusive target *sco5158*, which is not a target of ScbR or ScbR2 homodimer (Li et al., 2017). We observed that AvaR1/AvaR2 heterodimer and the AvaR1 and AvaR2 homodimers all bind to the same DNA probe *aveRp*_2_; however, further investigation is needed to test the possibility that AvaR1/AvaR2 heterodimer has exclusive targets as well.

Competitive EMSAs indicated that AvaR1 and AvaR2 homodimers compete for the same binding region. *In vivo* ChIP-qPCR experiments revealed that maximal DNA enrichment level of AvaR2, in comparison with control IgG, was ~10.5-fold for *aveRp*, ~23.3-fold for *acop*, ~8.4-fold for *avaR1p*, ~4.1-fold for *avaR2p*, and ~4.6-fold for *avaR3p* (Zhu et al., 2016). Corresponding values for AvaR1 were <2-fold for all five target genes, indicating that *in vivo* DNA-binding affinity of AvaR2 is stronger than that of AvaR1, and that the role of AvaR2 in regulating these common targets is dominant over that of AvaR1. AvaR2 showed stronger binding affinity for *aveRp* than for *avaR3p, avaR2p, acop*, or *avaR1p*. Its binding to *aveRp* was strongest at 24 h, and that to the other four target promoters was strongest at 48 h, with subsequent gradual decrease in binding strength during the fermentation process (Zhu et al., 2016). AvaR1 showed little difference in affinity among these five promoters; it bound to them at 24, 96, and 144 h, with somewhat higher binding strength at 24 h. The dynamic binding of AvaR1 and AvaR2 to the five targets was consistent with their expression profiles, i.e., maximal expression occurred at 24 h for AvaR1 and at 48 h for AvaR2; AvaR2 level decreased sharply after 96 h and was barely detectable from 120 h onward, whereas AvaR1 level was stable from 72 h onward.

**Figure 8:**
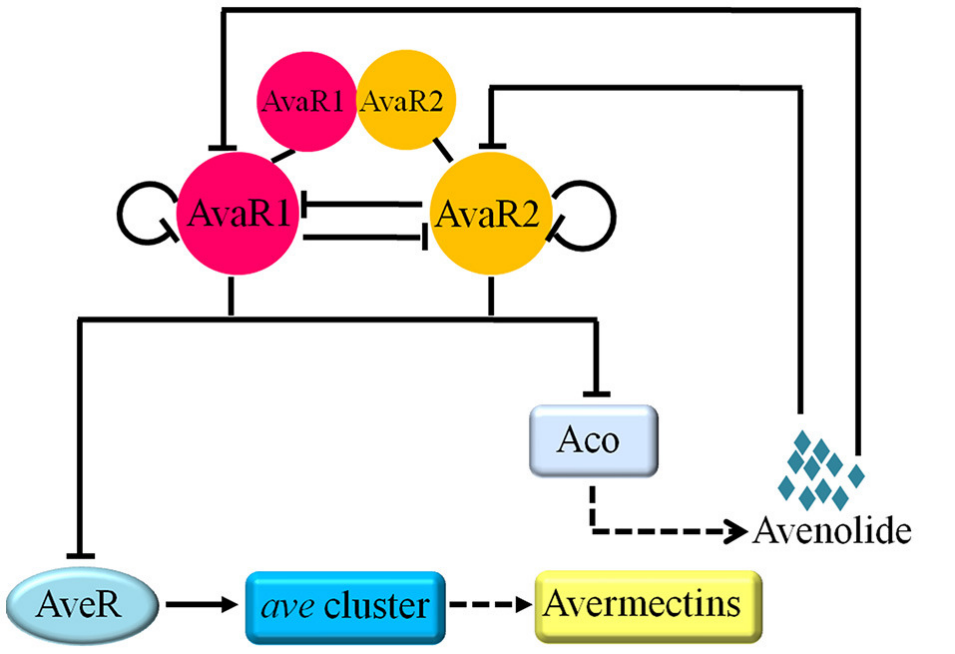
Working model for the regulatory roles of AvaR1 and AvaR2 in control of avenolide and avermectin production. Solid-line bars: direct repression. Solid-line arrow: direct activation. Dashed-line arrows: production of avermectin or avenolide.

Our findings on AvaR1 and AvaR2 expression and their regulatory relationship, taken together, indicate that they have a sequential cooperative mechanism for control of avenolide and avermectin production in response to avenolide signal (Figure 8). During the early growth phase of *S. avermitilis, aveRp* is repressed by AvaR1 and (preferentially) AvaR2 to strongly inhibit avermectin biosynthesis (24 h); AvaR1 (24 h) and AvaR2 (48 h) sequentially repress *aco* expression to strictly control avenolide level and avenolide synthesis remains at basal level during this period. When avenolide level exceeds a threshold value, its receptors AvaR1 and AvaR2 are sequentially released from *acop*, leading to sharply increased avenolide concentration which becomes sufficient to trigger avermectin biosynthesis by releasing AvaR1 and AvaR2 from *aveRp*. This concept is supported by our observation that avermectin production is usually detectable by HPLC after 48 h. After avenolide has performed its role in triggering avermectin production, it is presumably degraded by a yet-unknown mechanism. During the late growth stage (≥144 h), *acop* and *aveRp* are bound again mainly by AvaR1 rather than AvaR2, to avoid overproduction of avermectins and ensure appropriate avermectin concentration in cells. The repression of *acop* and *aveRp* by AvaR1 during late stage is advantageous in that the relatively low repression strength of AvaR1 ensures continuous, but not excessive, synthesis of avermectins.

Although AvaR1 and AvaR2 have common targets, they each also have exclusive targets, and their regulatory mechanisms on common target *aveT* are different. Thus, the regulatory functions of these two proteins differ somewhat. For example, cell growth is promoted by *avaR2* deletion (Zhu et al., 2016), but unaffected by *avaR1* deletion. These findings are consistent with general economic principles of metabolic regulation, i.e., a given microorganism does not contain two identical regulatory factors. The AvaR1 targets are involved in diverse cellular processes, indicating the pleiotropic effects of AvaR1 on *S. avermitilis* physiology. Elevated expression of AvaR1 target genes *leuD, nuoB1, rpmB1*, and *rpsQ* in ΔavaR1 may promote avermectin production by providing more precursors, increasing availability of energy, or enhancing protein synthesis, analogously to their effects in ΔavaR2 (Zhu et al., 2016). AveT acts as an activator of avermectin production. Therefore, elevated expression of *aveT* in ΔavaR1 may enhance avermectin production. The *cpdB* product converts 2′,3′-cyclic nucleotide to 3′-nucleotide, and increased *cpdB* expression may provide more precursors for ATP synthesis and thereby promote avermectin biosynthesis. Our findings that AvaR1 regulates *sig29* (whose homolog *sigI* in *S. coelicolor* is involved in osmotic stress) (Homerova et al., 2012) and *sav_3560* (one component of a two-component system) suggest that AvaR1 responds to extracellular stimuli as part of adaptation to complex natural environments. More extensive studies of regulatory roles of AvaR1 and AvaR2 in the future will provide a comprehensive picture of the cellular responses triggered by avenolide and other signals, and mediated by AvaR1 and AvaR2, in *S. avermitilis*.

## Author contributions

YW and JZ wrote the manuscript and designed the research. JZ performed experiments. ZC and JL contributed study materials.

### Conflict of interest statement

The authors declare that the research was conducted in the absence of any commercial or financial relationships that could be construed as a potential conflict of interest.
