# Supplementary material for: AvaR1, a Butenolide-Type Autoregulator Receptor in Streptomyces avermitilis, Directly Represses Avenolide and Avermectin Biosynthesis and Multiple Physiological Responses
Source: Front Microbiol. 2017 Dec 22;8:2577. doi: 10.3389/fmicb.2017.02577 (PMC5744401; doi:10.3389/fmicb.2017.02577)
Supplement: Supplementary file 1 [file Presentation1.PDF]

Supplemental Materials

AvaR1, a Butenolide-Type Autoregulator Receptor in *Streptomyces avermitilis*, Directly Represses Avenolide and Avermectin Biosynthesis and Multiple Physiological Responses

Jianya Zhu[1,2], Zhi Chen[1], Jilun Li[1] and Ying Wen[1]*

1. *State Key Laboratory of Agrobiotechnology and MOA Key Laboratory of Soil Microbiology, College of Biological Sciences, China Agricultural University, Beijing, China*
2. *Beijing Fisheries Research Institute, Beijing Key Laboratory of Fishery Biotechnology, Beijing, China*

*** Correspondence:**

Ying Wen

E-mail: wen@cau.edu.cn Phone: +86-10-62732715

Supplementary Figures:

FIGURE S1: Schematic strategy for *avaR1* deletion.

FIGURE S2: Effects of *avaR1* deletion and overexpression on morphological differentiation.

FIGURE S3: Avermectin yield of WT, ΔavaR1, and ΔavaR1/avaR1-3FLAG (ΔavaR1 bearing pSET152-avaR1-3FLAG) cultured in FM-I for 10 days.

FIGURE S4: Consensus sequence analysis of AvaR2 binding sites using the WebLogo program.

FIGURE S5: Confirmation of novel AvaR2 target genes.

FIGURE S6: Comparative avermectin yield in WT, ΔavaR1, ΔavaR2, and *avaR1 avaR2* double deletion mutants (ΔavaR1R2-1, ΔavaR1R2-2, ΔavaR1R2-3) cultured in FM-I for 10 days.

Supplementary Table:

TABLE S1: Primers used in this study.

Reference:

Zhu, J., Sun, D., Liu, W., Chen, Z., Li, J., and Wen, Y. (2016). AvaR2, a pseudo γ-butyrolactone receptor homologue from *Streptomyces avermitilis*, is a pleiotropic repressor of avermectin and avenolide biosynthesis and cell growth. *Mol. Microbiol*. 102, 562-578. doi: 10.1111/mmi.13479

FIGURE S1

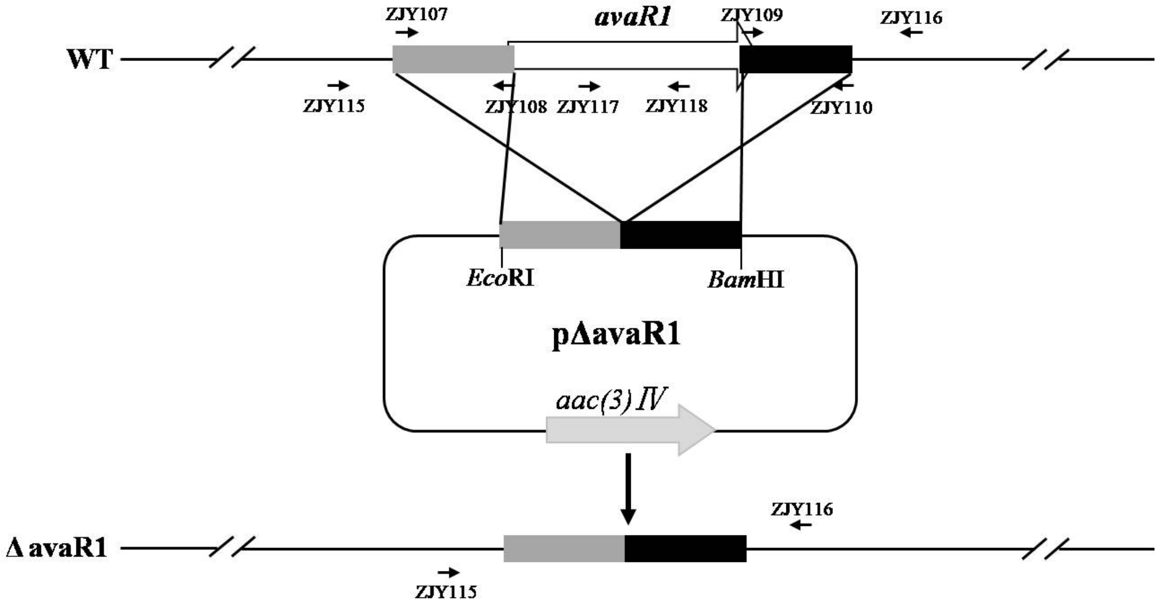

FIGURE S1 | **Schematic strategy for *avaR1* deletion.** Large arrows: genes and their directions. Small arrows: positions of primers. Rectangles: homologous exchange regions used for *avaR1* deletion.

FIGURE S2

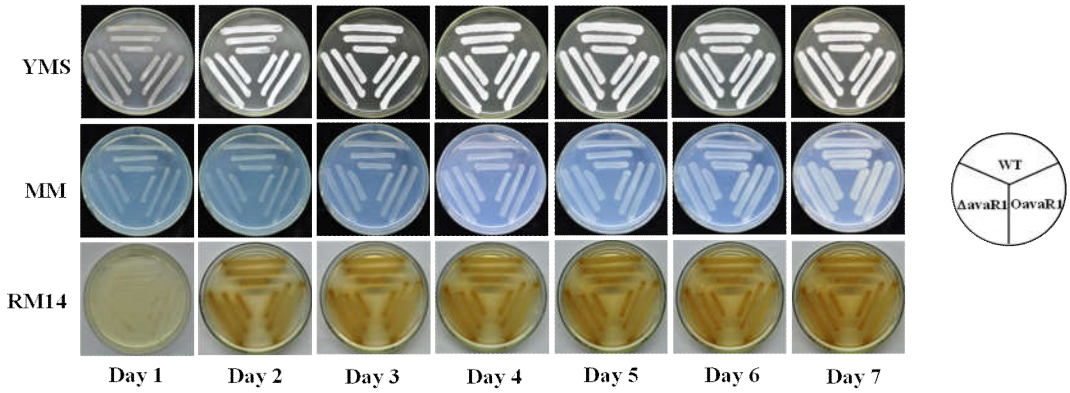

FIGURE S2 | Effects of *avaR1* deletion and overexpression on morphological differentiation. WT strain ATCC31267, *avaR1* deletion mutant ΔavaR1, and *avaR1* overexpression strain OavaR1 were grown on YMS, MM, or RM14 plates at 28°C, and photographed on days 1-7.

FIGURE S3

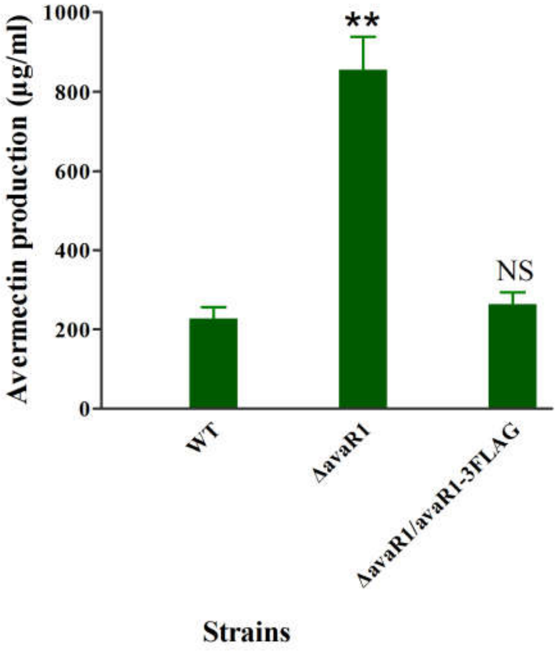

FIGURE S3 | Avermectin yield of WT, ΔavaR1, and ΔavaR1/avaR1-3FLAG (ΔavaR1 bearing pSET152-avaR1-3FLAG) cultured in FM-I for 10 days. NS, not significant; **, $P < 0.01$ for comparison with WT (Student's *t*-test).

FIGURE S4

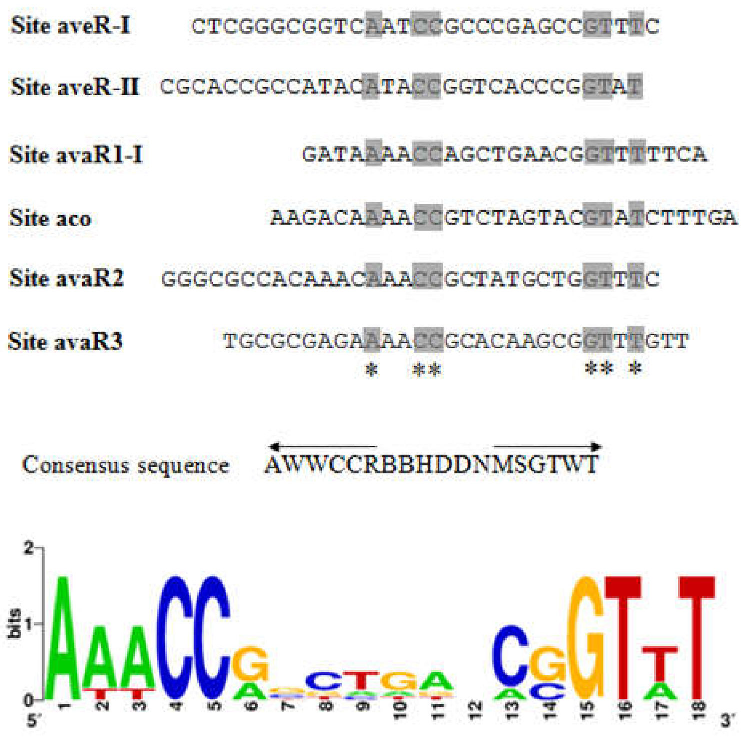

FIGURE S4 | Consensus sequence analysis of AvaR2 binding sites using the WebLogo program. Asterisks: consensus bases. Arrows: inverted repeats. Height of each letter is proportional to appearance frequency of corresponding base. (Zhu et al., 2016)

FIGURE S5

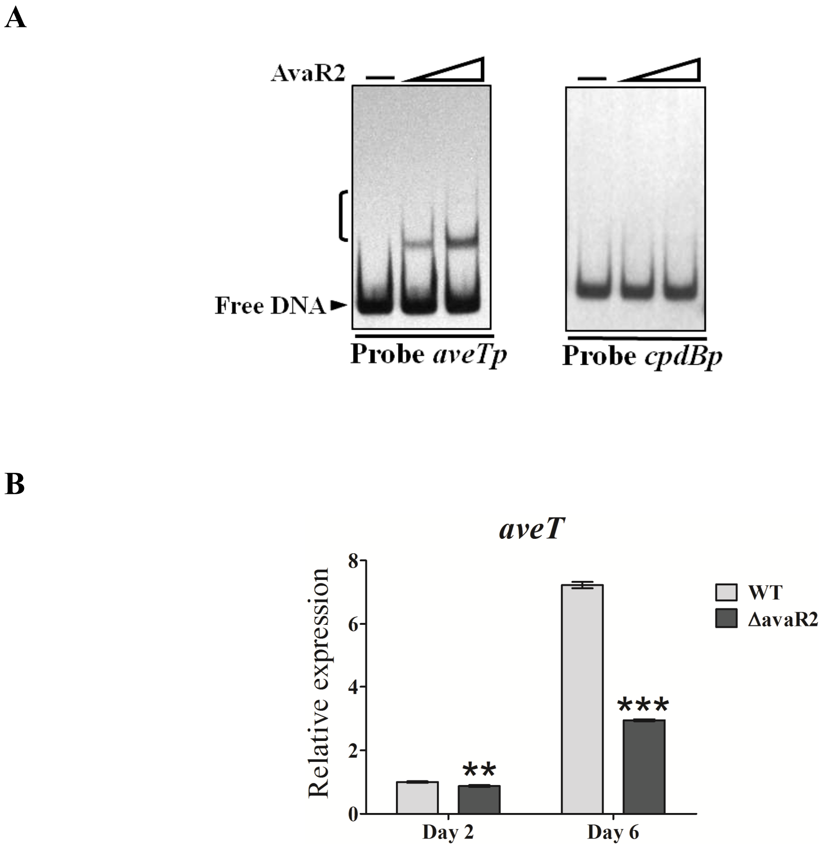

FIGURE S5 | Confirmation of novel AvaR2 target genes. (A) EMSAs of His_6-AvaR2 with probes *aveTp* and *cpdBp*. Lanes –: EMSAs without His_6-AvaR2. Lanes 2 to 3 contained 50 and 200 nM His_6-AvaR2, respectively. Each lane contained 0.3 nM labeled probe. Arrowheads: free probes. Brackets: AvaR2-DNA complexes. **(B)** qRT-PCR analysis of *aveT* in WT and ΔavaR2 (*avaR2* deletion mutant) grown in FM-I. Transcription level of *aveT* is expressed relative to WT value on day 2, defined as 1. **, $P < 0.01$; ***, $P < 0.001$ (Student's *t*-test).

FIGURE S6

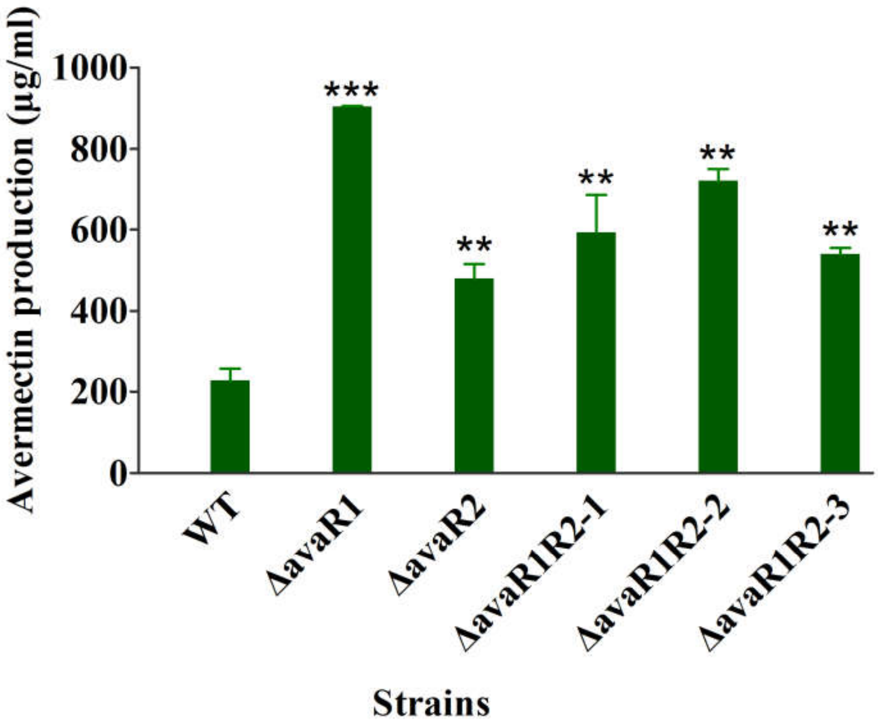

FIGURE S6 | **Comparative avermectin yield in WT, ΔavaR1, ΔavaR2, and *avaR1 avaR2* double deletion mutants (ΔavaR1R2-1, ΔavaR1R2-2, ΔavaR1R2-3) cultured in FM-I for 10 days.** Error bars: SD from three replicate experiments. **, $P< 0.01$; ***, $P< 0.001$ (Student's *t*-test).

TABLE S1 | Primers used in this study.

Primer purpose and primer	DNA sequence (5'-3')	Use
For gene deletion, complementation, and overexpression		
ZJY107	CGGAATTCTGCGGAAGAGCTGGAGGTC, *Eco*RI	Deletion of *avaR1* gene
ZJY108	CGACTCCCGCTGCTTCATGGAATCGTCTGCCGCGTCC	
ZJY109	GGACGCGGCAGACGATTCCATGAAGCAGCGGGAGTCG	
ZJY110	CGCGGATCCGCGTAGGGGCACTGGTTC, *Bam*HI	
ZJY115	GAGCTTCTCCCTGGCTTGG	Confirmation of *avaR1* deletion in ΔavaR1
ZJY116	TGCTTCACGTACTTCTGCCG	
ZJY117	GAGGTGTTCGACGAGGTGG	
ZJY118	CGACTCCCGCTGCTTCATG	
ZJY111	CGGAATTCGGGGAGCATTGAAAAACCGT, *Eco*RI	Complementation of *avaR1* in ΔavaR1
ZJY112	CGGGATCCCAGTCACTCCAACGTCCGT, *Bam*HI	
ZJY113	CCCAAGCTTGTGGCGCGGCAGGAGCGAG, *Hin*dIII	Overexpression of *avaR1* in *S. avermitilis*
ZJY114	GCTCTAGACAGTCACTCCAACGTCCGTGCC, *Xba*lI	
YRJ1	CGGAATTCGTGTCCGTTCGAGTGGCG, *Eco*RI	Amplification of *ermE*p* fragment
YRJ2	CCCAAGCTTGCTCACCGCTGGATCCTAC, *Hin*dIII	
ZJY121	GGAATTCGGGGAGCATTGAAAAACCGTTC, *Eco*RI	Complementation of *avaR1* in ΔavaR1 with 3×FLAG-tagged AvaR1
ZJY122	CCCAAGCTTCTCCAACGTCCGTGCCG, *Hin*dIII	
ZJY45	CCCAAGCTTGGAGGTGGCATGGACTAC, *Hin*dIII	Amplification of 3×FLAG fragment
ZJY46	GCTCTAGATCCGGTTGACCCCTATTTATC, *Xba*lI	
ZJY135	CGGGATCC ACGAAACAGGAACGCGC, *Bam*HI	Overexpression of GST-AvaR2 in *E. coli*
ZJY136	CGGAATTCTACGGCTCATCCGGCGGT, *Eco*RI	
For EMSA		
ZJY15	CAGGGAGGTGCCGGGGG	Probe *avaR2p*
ZJY16	GCGGAAGGCGGCTGACC	
ZJY17	CGCCCTCTCACAAACCACTC	Probe *acop*
ZJY18	GAGCTTCTCCCTGGCTTGG	
ZJY19	GCCACACGCACCCTCCG	Probe *avaR1p*
ZJY20	GGGAGTTGGG GGTGAGATATGTG	
ZJY21	ACTGATGCGCGAGAAAAC	Probe *avaR3p*
ZJY22	GGACTCGATCAAGGCGTT	
ZJY23	TGACGCCTGGTCCTCCG	Probe *aveRp*
ZJY24	GTGCTGCAACACCGAATGG	
LS58	ATGGTCGGGAACCTCCGCAA	Probe *aveA1p*
LS59	CTGTGTCCTCACCGCTAGGC	
LS36	TCAACCACCGTCATGTAGGG	Probe 1
LS37	GGCAAGCTTTTCGACACAAG	
ZJY55	CCTCATCGCCTTCGTCAT	Probe *pstBp*
ZJY56	CCTTGTGGGAGCCGTAGT	

ZJY57	GCTCTTCGTGCTCACCGT	Probe *nuoB1p*
ZJY58	GTCGTCAGCAGGAATCCG	
ZJY59	CACAGGGTGGCGGAGAGG	Probe *folP2p*
ZJY60	TCATGCGTCCAGCCTAGGC	
ZJY61	CCGCTGGAGTCTGAGAAG	Probe *leuDp*
ZJY62	CGGTTGAGGATGAAGGAC	
ZJY63	GAACTGGGTGACGAGGAG	Probe *rpsQp*
ZJY64	TTGTCCATCTTGTCGCTG	
ZJY65	AGTCGTTGTATGCTGCTCC	Probe *rpmBp*
ZJY66	GAGTGCGAGATGTTGTTGC	
ZJY67	CGAACAACTTACCCAAATCG	Probe *sig29p*
ZJY68	GATGCGTGCT CCAGGTTT	
ZJY69	CCTCGCCGTTGATCTCACC	Probe *amfCp*
ZJY70	CCCTCGACCGGATTGTGG	
ZJY71	GGCACGACAACCACCAGTT	Probe *1230p*
ZJY72	CATGACCACG GCCAAGGC	
ZJY73	GCGTTGCACTCCTCGTCG	Probe *2051p*
ZJY74	TGCGGTGACCCCCATCTC	
ZJY75	TTGTGCCCTGTGTGTGCG	Probe *3560p*
ZJY76	ATGCCTCCCAGCCTACGG	
ZJY89	TCACGGAATGTCTGCCAC	Probe *wbpAp*
ZJY90	GCCCTCGTCCCAGTAGAAG	
ZJY91	CCATCAACCAGAA GAACGAG	Probe *fadE22p*
ZJY92	GCACTGTCAAGGTCACGG	
ZJY93	TCGCCATTCGGGTCAGTC	Probe *ileSp*
ZJY94	ATCCACGCTTCTCGCTCC	
ZJY95	TTCAACTGACCGTCCCGC	Probe *melC2p*
ZJY96	AGGAAGGCGAACGATGGC	
ZJY123	GCTGACGGTGATGCCTAT	Probe *cpdBp*
ZJY124	TTCAAGAACTTCCGACGGT	
LWS13	TCGGACTCGGGGTTCACCT	Probe *aveTp*
LWS14	CTCGGGCGTGATCCGACT	
ZJY133	GCCATACATACCGGTCACCC	Probe $aveRp_2$
ZJY134	GGGTAGTGTCAAGGTCGCTG	
For DNase I footprinting		
FAM-ZJY19	GCCACACGCACCCTCCG	*avaR1* promoter region
ZJY20	GGGAGTTGGGGGTGAGATATGTG	
FAM-ZJY25	CGCCCTCTCACAAACCACTCG	*aco* promoter region
ZJY11	GCCGGCCTCGAACGTCG	
FAM-ZJY12	TGCACATCAAGAAGGGCG	*avaR3* promoter region
ZJY22	GGACTCGATCAAGGCGTT	
FAM-ZJY13	TACGCCCGAGGAATGTGTC	*avaR2* promoter region
ZJY14	CGCCTGTGTGTAGCCCTG	
FAM-ZJY23	TGACGCCTGGTCCTCCG	*aveR* promoter region

ZJY26	CTCCCTGCAT GATGTTCTTATTTCT	
For qRT-PCR		
ZJY27	CCACTACAACCTGTTCCTCGG	*aco* ORF
ZJY28	GTTGCCGTGCTCCAGTTCG	
ZJY29	TCTGGTCAGCCGCCTTCCG	*avaR2* ORF
ZJY30	AGCGGATGAG GGCGTGGC	
ZJY31	CCCCCATCAGCAACGGAGC	*avaR3* ORF
ZJY32	ATCGCGCGTTCCTGTCTG	
ZJY129	TTGGTCTTTTCCCGCTGGTC	*avaR1* ORF
ZJY130	CGAATGGCTCGCTCCTGC	
ZJY35	CAGAGGGAGTCGGAGCAGC	*cyp17* ORF
ZJY36	CGGCAGCTCCACCTCCAC	
16S11	AGCGGAGCATGTGGCTTAAT	16S *rRNA* ORF
16S22	ACGTATTCACCGCAGCAATG	
GJ97	CAGAAGAACTCACGCTCGTC	*aveR* ORF
GJ98	ACTCTTTCCACAGCCCATTC	
GJ99	CGGACAGGACTACGCACTTC	*aveA1* ORF
GJ100	ACGAGATACGACCGGAGATG	
ZJY79	CTTCGGCATGGAGGTCTTC	*nuoB1* ORF
ZJY80	CATCTTCTGGCTGACCCG	
ZJY81	GTCATGGGGGTCGTCAAC	*folP2* ORF
ZJY82	GGTGACCAGGTCGAGTCC	
ZJY83	TCCAGAACTACGGCTTCAAGG	*leuD* ORF
ZJY84	GAACCACTGTGAGCAGGCC	
ZJY85	TCAGCGACAAGATGGACAAG	*rpsQ* ORF
ZJY86	CCATGAGGAGGACACGGT	
ZJY87	CTGCCAACTGCGACGTC	*rpmB* ORF
ZJY88	GCACACGCTGGATGTTCG	
ZJY99	GTATGTCGTTCGTCGGGAT	*sav_3560* ORF
ZJY100	GGTGATGGACAGGAAGGG	
ZJY103	GCGAACCGATGGAAGACAT	*sig29* ORF
ZJY104	CACGGAAGAAACGCTTGATC	
ZJY125	CGAGTCGCAGTGCCGTTT	*cpdB* ORF
ZJY126	GCACAGCCACCTTCACCG	
LWS25	CCGTGTCGTTCGAAGCA	*aveT* ORF
LWS26	GAGTACAGCTCGGCCTC	
SD218A	GCCGACCTCAGCTATGTG	*amfC* ORF
SD218B	GAAAGCCGCTCCATGAC	
For ChIP-qPCR		
ZJY37	CCGCTCGAGTGACGCCTGGTCCTCCG	*aveR* promoter DNA
ZJY38	GTGCTGCAACACCGAATGG	
ZJY47	TCGGCGAAGTGGTGACGG	*avaR2* promoter DNA
ZJY48	GGCGCGTTCCTGTTTCGT	
ZJY49	CACTGATGCGCGAGAAAAC	*avaR3* promoter DNA

ZJY50	CGTTCCTGTCTGACCTGCTT	
ZJY51	CAGCGGGAAAAGACCAAG	*avaR1* promoter DNA
ZJY52	TCAGCTGGTTTTATCGTCCC	
ZJY53	CAAAAACACATATCTCACCCCC	*aco* promoter DNA
ZJY54	GGAAGAGCTGGAGGTCAAAG	
ZJY131	CCCTAAGAAATGAACACCCTTGG	*aveA1* promoter DNA
ZJY132	CGAAGAATGAAAGGAGCGCG	
For reporter system		
ZJY39	CCGCTCGAGTGACGCCTGGTCCTCCG, *Xho*I	Amplification of *aveR*
ZJY40	CGGGATCCCTCCCTGCATGATGTTCTTATTTCT, *Bam*HI	promoter in pOaveRlux
ZJY127	GCTGGTGCGGAGGGTGC	Amplification of *avaR1* ORF
ZJY128	CGGGATCCCACGCCGGACAGTCACTC, *Bam*HI	and RBS in pAvaR1
